# E-ROBOT: a dimension-free method for robust statistics and machine learning via Schrödinger bridge

**DOI:** 10.1007/s11222-026-10914-9

**Published:** 2026-06-06

**Authors:** Davide La Vecchia, Hang Liu

**Affiliations:** 1 Geneva School of Economics and Management, University of Geneva, Bld. du Pont d’Arve, Geneva, CH-1211 Switzerland; 2 Department of Statistics and Finance, School of Management, University of Science and Technology of China, Jinzhai Rd, Hefei, 230026 Anhui Province China

**Keywords:** Optimal transport, Curse of Dimensionality, Goodness-of-Fit Test, Barycenters, Gradient flow, Outliers

## Abstract

We propose the Entropic-regularized Robust Optimal Transport (E-ROBOT) framework, a novel method that combines the robustness of ROBOT with the computational and statistical benefits of entropic regularization. We show that, rooted in the Schrödinger bridge problem theory, E-ROBOT defines the robust Sinkhorn divergence $\overline{W}_{\varepsilon ,\lambda }$, where the parameter λ controls robustness and ε governs the regularization strength. Letting $n\in \mathbb {N}$ denote the sample size, a central theoretical contribution is establishing that the sample complexity of $\overline{W}_{\varepsilon ,\lambda }$ is $\mathcal {O}(n^{-1/2})$, thereby avoiding the curse of dimensionality that plagues standard ROBOT. This dimension-free property unlocks the use of $\overline{W}_{\varepsilon ,\lambda }$ as a loss function in large-dimensional statistical and machine learning tasks. With this regard, we demonstrate its utility through four applications: goodness-of-fit testing; computation of barycenters for corrupted 2D and 3D shapes; definition of gradient flows; and image colour transfer. From the computation standpoint, a perk of our novel method is that it can be easily implemented by modifying existing (Python) routines. From the theoretical standpoint, our work opens the door to many research directions in statistics and machine learning: we discuss some of them.

**Supplementary Information:**

The online version contains supplementary material available at https://doi.org/10.1007/s11222-026-10914-9.

## Introduction

### Related work

The notion of the Schrödinger bridge problem (SBP) originates in Schrödinger (1931), when Schrödinger investigated the most likely evolution of a cloud of independent Brownian particles subject to boundary conditions.

Intuitively, the SBP searches for the most likely joint distribution of initial and final cloud of particles that is consistent with the observed endpoint results (namely, the original and target measures). This entropy-minimization problem exhibits a profound unity across seemingly distinct fields, including statistical and quantum physics, probability, information theory, statistics, machine learning, and artificial intelligence. We refer the reader to Rigollet and Weed (2018); Peyré and Cuturi (2019); Wang et al. (2021); Liu et al. (2022); Bunne et al. (2023); Pooladian and Niles-Weed (2025) and references therein.

One of the main attractive features of the SBP is its link to optimal transport (OT) problem; see Léonard (2013). This connection yields two significant advantages. The first advantage is theoretical: the sample complexity of the Sinkhorn divergence, which results from the combination of SBP and OT, scales at a rate better than that of typical unregularized OT distances, which suffer from the curse of dimensionality. Essentially, the problem with standard OT is that Wasserstein distance computed between two samples converges very slowly to its population counterpart; see Genevay et al. (2019). Moreover, Feydy et al. (2019) show that entropic-regularized OT (E-OT) interpolates between the Wasserstein distance and the Maximum Mean Discrepancy (MMD, see e.g. Sriperumbudur et al. (2011)). Specifically, it preserves the appealing geometric properties of OT losses, and, at the same time, it benefits from the low sample complexity of MMD norms. The second advantage is computational: the Sinkhorn algorithm accelerates the computation of an approximate transport plan, significantly expanding the range of OT applications, particularly in machine learning; see Cuturi (2013); Peyré and Cuturi (2019).

However, a well-known limitation of both OT and E-OT is their sensitivity to anomalous records and their requirement of finite moments—both issues stem from the use of an unbounded cost function in the transportation cost definition; see e.g. Mukherjee et al. (2021); Nietert et al. (2022); Ma et al. (2025). To address these problems, Ma et al. (2025) introduce robust optimal transport (ROBOT) and study its statistical properties, proving its robustness to outliers. Despite these advantages and its good performance in many statistical and machine learning tasks, ROBOT still suffers from the curse of dimensionality and exhibits multi-scale behavior. Similarly to OT-based distances (see e.g. Weed and Bach (2019)), the sample complexity of ROBOT-based distances depends on the data dimension; see Theorem 10 in Ma et al. (2025).

Given the widespread use of OT in time series analysis, statistics, and machine learning over the past ten years (see, among others, Panaretos and Zemel (2019); Peyré and Cuturi (2019); Hallin (2022); La Vecchia et al. (2022); Hallin et al. (2022, 2023); Chewi et al. (2025); Hallin and Liu (2024); Barigozzi et al. (2025)), this limitation significantly restricts the applicability of ROBOT-based methods. In the OT literature, however, solutions exist to mitigate the curse of dimensionality. For instance, the sliced-Wasserstein metric projects higher-dimensional distributions into one-dimensional representations and computes the distance as a functional of the Wasserstein distances between these projections; see e.g. Kolouri et al. (2017); Peyré and Cuturi (2019) for a review. Alternatively, Gaussian-smoothed OT applies isotropic Gaussian smoothing to the original measures, alleviating the curse of dimensionality while preserving structural properties of the Wasserstein distance; see Goldfeld and Greenewald (2020); Nietert et al. (2021). Unfortunately, to the best of our knowledge, no robustness guarantees are available for these methods: although applicable in high dimensions, they still rely on unbounded cost functions and thus remain sensitive to outliers. This limits their applicability in the presence of anomalous records or when distributions lack finite moments.

### Contribution, structure, and preview

*Contribution.* In this paper, we propose a method that addresses both the dimensionality and robustness issues of OT. Our approach bridges a gap in the OT literature within machine learning while also contributing to multivariate and robust statistics. We examine both theoretical and computational aspects and illustrate the ease of implementation of our methodology. Our contribution can be summarized as follows.

(i) We develop the mathematical foundation of the E-ROBOT framework in a self-contained manner, organizing key properties into several propositions and one theorem for clarity and ease of reference. An elegant feature of our development is that some results follow from existing theory–requiring only minor adjustments to propositions and proof strategies once the truncated cost is introduced (see Propositions 1, 2, 5, 7, 8, and to some extent Propositions 4, 6, and 12). This parsimony underscores how seamlessly E-ROBOT integrates into and leverages the broader theoretical landscape of E-OT. In contrast, other results–notably the global regularity of the potentials in Proposition 3 and the dimension-free sample complexity in Theorem 9 and Corollary 10–are not direct consequences of existing theorems and require more elaborate arguments specific to E-ROBOT, thereby representing a genuine theoretical contribution.

(ii) We investigate the methodological, computational, and implementation aspects of E-ROBOT, illustrating its versatility across a range of statistical and machine-learning tasks. Regarding computational aspects, a key advantage of our method is its straightforward integration into existing algorithms–a feature that renders E-ROBOT both simple to implement and effective in practice.

The contribution of the paper lies in both (i) and (ii): the manuscript is organized to highlight this dual value, balancing mathematical developments with statistical and computational considerations.

*Structure.* To assist the reader, we provide an overview of the structure of the paper. In §2 and §3, building on SBP theory, we introduce E-ROBOT, which combines the robustness of ROBOT with the computational and statistical benefits of entropic regularization. In §3, we derive the main theoretical and methodological properties of E-ROBOT. Specifically, we provide the functional form of its potentials and their properties (Propositions 1, 3, 4), its dual formulation (Proposition 2), and the convergence of the transport plan as the regularization vanishes (Proposition 5) and as the sample size increases (Proposition 6). We show how E-ROBOT defines a truncated robust Sinkhorn divergence $\overline{W}_{\varepsilon ,\lambda }$ that metrizes convergence in law (Proposition 7), and how the Schrödinger potentials induce a Bregman-type divergence (Proposition 8). One of our main contributions is Theorem 9 and Corollary 10, establishing the dimension-free sample complexity of $\overline{W}_{\varepsilon ,\lambda }$. We also justify the use of E-ROBOT as a loss function for statistical inference and machine-learning applications (Proposition 12). While some results follow from SBP and OT theory, others require careful adaptation to the truncated-cost setting, revealing additional implications such as the new MMD connections discussed after Proposition 7 and after Corollary 10 in Remark 11.

In §4, we illustrate the applicability and performance of E-ROBOT in several statistical and machine-learning settings. Readers primarily interested in computational aspects may proceed directly to that section. Our experiments demonstrate that implementing E-ROBOT requires only minor modifications to existing Python and R libraries: one replaces the unbounded cost matrix of standard OT with the bounded cost matrix in (7). This yields ready-to-use tools that are reliable in the presence of outliers and in high dimensions. Furthermore, our experiments show that E-ROBOT enables inference in high dimensions even when the underlying distribution lacks finite moments – such as multivariate *t*-distributions with small degrees of freedom (*df*) – a regime where standard OT and entropic OT (e.g. $W_p$) fail due to moment requirements.

In §5, we discuss future research directions that naturally extend the E-ROBOT framework. We highlight potential theoretical developments in robust divergence-based parametric inference (noting also potential connections to differential privacy) and methodological tasks such as the joint optimization of λ and ε. Although beyond the scope of this paper, highlighting these pathways provides a foundation for future study.

**Fig. 1:**
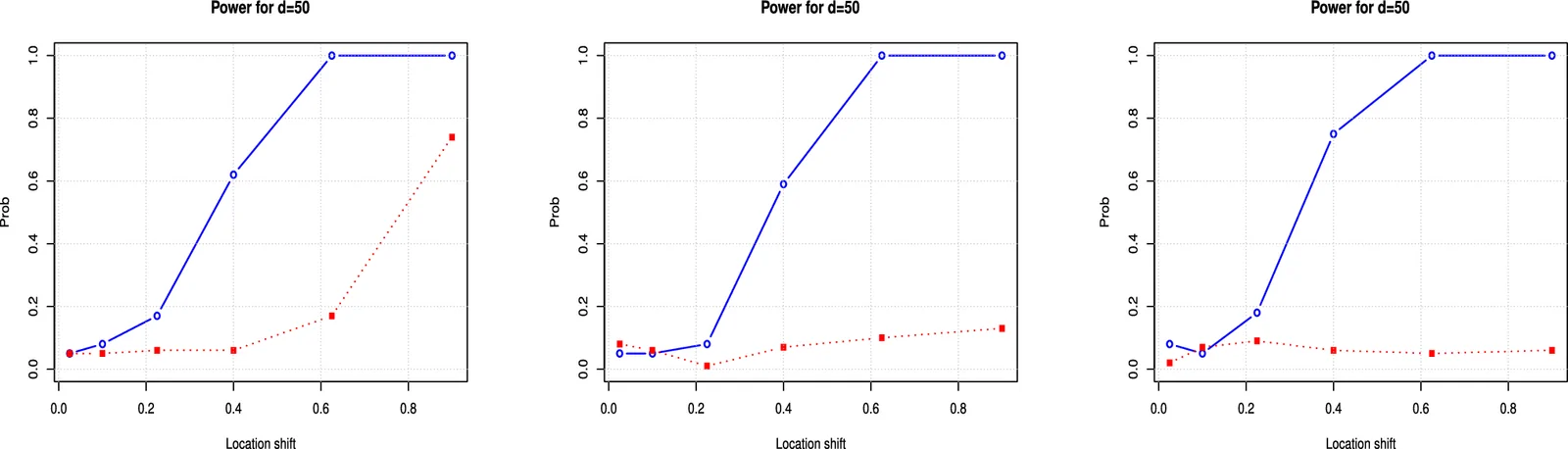
Power curves for testing the null hypothesis of a *d*-variate *t*-distribution with different degrees-of-freedom (*df*), location zero, and identity scale matrix. The continuous (blue) curve with circles corresponds to our new test statistics based on $\overline{W}_{\varepsilon ,\lambda }$, with ε=0.05 and λ=3; the dotted (red) curve with squares corresponds to the test statistics based on $W_1$. Left plot is for df=3, middle plot for df=2, right plot for df=1

*Preview.* To illustrate the advantages of E-ROBOT and the limitations of existing methods, we preview one representative result. Figure 1 presents outcomes for a simple hypothesis test of distributional equality; see Hallin et al. (2021). We compare our $\overline{W}_{\varepsilon ,\lambda }$-based test (continuous blue curve) with the $W_1$-based test of Hallin et al. (2021) (dotted red curve); see §4.1 for details. In a high-dimensional setting (d=50), we simulate from *t*-distributions with varying degrees of freedom. At a 5% level, the $W_1$ test has low power for df=1,2, improves somewhat at df=3 (where first and second moments exist), but is consistently outperformed by our test across all *df* and alternatives. This shows that standard OT-based procedures may struggle with moment conditions and dimensionality even in simple scenarios.

Appendix A and Appendix B in the Supplementary Material contain all proofs (with details and remarks) and additional numerical results.

## SBP, ROBOT, and E-ROBOT

In §2.1 and §2.2 we recall the key theoretical aspects of SBP and ROBOT that are needed for our developments; we refer to Léonard (2013) and to Ma et al. (2025) for more details. Then, in §2.3 we explain how SBP and ROBOT can be blended to obtain the E-ROBOT.

### SBP

Let (X,μ) and (Y,ν) be separable probability spaces, and let $\mathcal {P}(X)$ denote the set of all probability measures on *X*. Let $R \in \mathcal {P}(X \times Y)$ be a reference measure. The goal is to find a coupling $\pi ^* \in \Pi (\mu , \nu )$ minimizing the relative entropy with respect to *R*:

1 $$\begin{aligned} \pi ^* = \arg \min _{\pi \in \Pi (\mu , \nu )} H(\pi \Vert R), \end{aligned}$$

where $\Pi (\mu , \nu )$ is the set of couplings with fixed marginals μ and ν and $H(\pi \Vert R) = \mathbb {E}_\pi [ \ln (\pi / R)]$ denotes the relative entropy (or Kullback-Leibler divergence) of π with respect to R. If $\Pi _{\textrm{fin}}(\mu , \nu ):= \{\pi \in \Pi (\mu , \nu ): H(\pi \Vert R) < \infty \} \ne \emptyset$, then there exists a unique minimizer $\pi ^*$. When *R* is absolutely continuous with respect to $\mu \otimes \nu$ (the product measure), the optimal $\pi ^*$ satisfies

2 $$\begin{aligned} \frac{d\pi ^*}{dR} = e^{\varphi ^*(x) + \psi ^*(y)} \quad R\text {-a.s.}, \end{aligned}$$

for some measurable functions $\varphi ^*: X \rightarrow \mathbb {R}$ and $\psi ^*: Y \rightarrow \mathbb {R}$, called Schrödinger potentials. These are unique up to an additive constant. Moreover, let $c: X \times Y \rightarrow (-\infty , \infty ]$ be a function and define

3 $$\begin{aligned} e^{-c(x, y)} = \frac{dR}{d(\mu \otimes \nu )}(x, y). \end{aligned}$$

Then, the optimal potentials $(\varphi ^*, \psi ^*)$ solve the Schrödinger system:

4 $$\begin{aligned} \varphi ^*(x)&= -\ln \int _Y e^{\psi ^*(y) - c(x, y)} \, \nu (dy), \end{aligned}$$

5 $$\begin{aligned} \psi ^*(y)&= -\ln \int _X e^{\varphi ^*(x) - c(x, y)} \, \mu (dx). \end{aligned}$$

We notice that the Schrödinger potentials $\phi ^*, \psi ^*$ satisfy the fixed-point equations in (4) and (5), which correspond to a SoftMax (log-sum-exp) operation. We will come back to this aspect in §2.3.

### ROBOT

The ROBOT framework, as defined in Mukherjee et al. (2021), modifies the classical Kantorovich formulation of OT by incorporating robustness to outliers via a total variation (TV) regularization. The primal formulation introduces an auxiliary perturbation *s* to the source measure μ, leading to the constrained problem:

$$\begin{aligned}&\min _{\pi , s} \int _X \int _Y c(x,y) \pi (x,y) dx dy + \lambda \Vert s\Vert _{\textrm{TV}} \\&\quad \text {s.t.} \quad \int _Y \pi (x,y) dy = \mu (x) + s(x), \\&\quad \int _X \pi (x,y) dx = \nu (y), \; \int _X s(x)dx = 0, \end{aligned}$$

where λ>0 controls the influence of outliers and c(x,y)=d(x,y), with $d(x,y)=\Vert x-y\Vert$ being the Euclidean distance. This formulation effectively eliminates data points *x* such that μ(x)+s(x)=0, identifying them as outliers. A computationally efficient equivalent formulation is given by:

$$\begin{aligned} \inf \left\{ \int _{{X} \times {Y}} c_{\lambda }(x,y) \, d\pi (x,y) : \pi \in \Pi (\mu ,\nu ) \right\} , \end{aligned}$$

with $c_{\lambda }(x,y):= {\tilde{d}}_{\lambda }(x,y):= \min \{ d(x,y), 2\lambda \}$, which replaces the original cost c=d with a trimmed version $c_\lambda$ that bounds the transport cost and introduces robustness. Ma et al. (2025) illustrate that the dual formulation of ROBOT, unlike classical OT, imposes a bounded range constraint on the Kantorovich potential ψ:

$$\begin{aligned} &  \sup \left\{ \int \psi \, d\mu - \int \psi \, d\nu : \psi \in \mathcal {C}_b({X}), \; |\psi (x) - \psi (y)|\right. \\  &  \left. \quad \ \le d(x,y), \; \textrm{range}(\psi ) \le 2\lambda \right\} , \end{aligned}$$

where for the sake of notation we omit the integration domain and $\mathcal {C}_b$ denotes the set of bounded and continuous functions. Moreover, by noticing that ${\tilde{d}}_{\lambda }(x,y)$ is a metric, one can construct the robust Wasserstein distance:

6 $$\begin{aligned} W^{(\lambda )}(\mu , \nu ) := \inf _{\pi \in \Pi (\mu ,\nu )} \int c_{\lambda }(x,y) d\pi (x,y), \end{aligned}$$

which is shown to be a proper metric on the space of probability measures $\mathcal {P}(X)$. Unlike standard Wasserstein distances, $W^{(\lambda )}$ remains well-defined for all probability measures, even without moment conditions, making it suitable for robust inference for heavy-tail data distributions. Additionally, $W^{(\lambda )}$ is continuous and monotonically non-decreasing with respect to $\lambda \in [0,\infty )$, and, if $W_1(\mu ,\nu )$ exists, $\lim _{\lambda \rightarrow \infty } W^{(\lambda )}(\mu ,\nu ) =W_1(\mu ,\nu ).$ For a Polish space (*X*, *d*) , if we suppose that $\mu _k$, (resp. $\nu _k$) converges weakly to μ, (resp. ν) in $\mathcal {P}(X)$ as $k \rightarrow \infty$, then $W^{(\lambda )}(\mu _k,\nu _k) \rightarrow W^{(\lambda )}(\mu ,\nu )$. Finally, Theorem 10 in Ma et al. (2025) proves that the sample complexity of $W^{(\lambda )}$ is:

$$ \mathbb {E}[W^{(\lambda )}(\mu ,{\hat{\mu }}_n)] = {\left\{ \begin{array}{ll} \mathcal {O}(n^{-1/2}) & \text { when }d=1\hspace{5.0pt},\\ \mathcal {O}(\frac{\ln n}{n^{1/2}}) & \text { when }d=2\hspace{5.0pt},\\ \mathcal {O}(n^{-1/d}) & \text { when }d\geqslant 3, \hspace{5.0pt}\end{array}\right. } $$

which implies that ROBOT suffers from the curse of dimensionality: for d>2 the dimension of the underlying space has an impact on the mean rate.

### E-ROBOT

Let the product measure $P= \mu \otimes \nu \in \mathcal {P}(X \times Y)$ be the reference measure. Then the entropic optimal transport problem with regularization parameter ε>0 is defined via the minimization over $\pi \in \Pi (\mu , \nu )$ of

$$ C_\varepsilon (\mu , \nu , c, \pi ) := \int c(x, y) \, d\pi (x, y) + \varepsilon H(\pi \Vert P). $$

To proceed further, let us consider the ROBOT cost function $c_\lambda$. For ε>0, we define the rescaled and truncated cost function

7 $$\begin{aligned} \varepsilon ^{ -1} c_\lambda (x,y) = \varepsilon ^{ -1} \min (d(x,y), 2\lambda ), \end{aligned}$$

which allows to define the entropic regularized ROBOT (E-ROBOT) problem. Omitting the arguments for the sake of notation, we introduce the truncated Laplacian kernel

8 $$\begin{aligned} k_{\varepsilon ,\lambda } = e^{- \varepsilon ^{-1} c_\lambda } \end{aligned}$$

(more generally, this is a Gibbs kernel) and define the reference (Gibbs) joint distribution

$$\begin{aligned} dR_\varepsilon := \frac{1}{\beta }k_{\varepsilon ,\lambda } dP, \quad \text {where } \beta := \int k_{\varepsilon ,\lambda } dP. \end{aligned}$$

For any $\pi \in \mathcal {P}(X \times Y)$ with π absolutely continuous w.r.t. $R_\varepsilon$, namely $\pi \ll R_\varepsilon$, we have the equalities

$$\begin{aligned} H(\pi \Vert R_\varepsilon )= &  \int \ln \left( \frac{d\pi }{dR_\varepsilon } \right) d\pi = \int \ln \left( \frac{d\pi }{dP} \frac{d P}{dR_\varepsilon } \right) d\pi \\= &  \int \ln \left( \frac{d\pi }{dP} \right) d\pi + \int \ln \left( \frac{d P}{dR_\varepsilon } \right) d\pi \\= &  H(\pi \Vert P) + \ln \beta + \int \varepsilon ^{-1} c_\lambda d \pi , \end{aligned}$$

so

9 $$\begin{aligned} C_\varepsilon (\mu , \nu , c_\lambda , \pi ) &  = \int c_\lambda \, d\pi + \varepsilon H(\pi \Vert P)\nonumber \\  &  = \varepsilon H(\pi \Vert R_\varepsilon ) - \varepsilon \ln \beta . \end{aligned}$$

The E-ROBOT problem is to minimize $C_\varepsilon$ in (9), that is

10 $$\begin{aligned} \inf _{\pi \in \Pi (\mu , \nu )} C_\varepsilon (\mu , \nu , c_\lambda , \pi ) = \inf _{\pi \in \Pi (\mu , \nu )} \varepsilon H(\pi \Vert R_\varepsilon ) - \varepsilon \ln \beta , \end{aligned}$$

which is a (static) Schrödinger bridge problem for $R_\varepsilon$, similarly to the problem stated in (1). When ε>0, the E-ROBOT problem is strongly convex, so that the optimal plan is unique. Then, the next propositions can be proved by making use of the specific form of $c_\lambda$ along the same lines as in Thm. 4.2 and Thm. 4.7 in Nutz (2021), to which we refer for the mathematical detail.

#### Proposition 1

Let $c_\lambda$ be the ROBOT cost function and let (X,μ) and (Y,ν) be separable. Then:

(i) there is a unique minimizer $\pi _{\varepsilon }^* \in \Pi (\mu , \nu )$ for the E-ROBOT problem in (10);
(ii) There exist measurable functions $\varphi ^* : X \rightarrow \mathbb {R}$, $\psi ^* : Y \rightarrow \mathbb {R}$ (those are potentials which in fact depend on both ε and λ) such that
  $$ \frac{d\pi _{\varepsilon }^*}{d(\mu \otimes \nu )} = e^{\varphi ^* + \psi ^* - \varepsilon ^{-1} c_\lambda } \quad \mu \otimes \nu \text {-a.s.} $$
  The potentials $\varphi ^*, \psi ^*$ are unique up to an additive constant, $\varphi ^* \in L^1(\mu )$, $\psi ^* \in L^1(\nu )$ and
  11 $$\begin{aligned} \varphi ^{*}(x)= &  -\varepsilon \ln \int e^{ \psi ^*(y) - \varepsilon ^{-1} c_\lambda (x,y)} \nu (dy) \ \ \mu -\text {a.s.} \end{aligned}$$
  12 $$\begin{aligned} \psi ^{*}(y)= &  -\varepsilon \ln \int e^{ \varphi ^*(x) - \varepsilon ^{-1} c_\lambda (x,y)} \mu (dx) \ \ \nu -\text {a.s.} \end{aligned}$$
  Conversely, if ${\bar{\pi }} \in \Pi (\mu , \nu )$ admits a density of the form
  $$ \frac{d{\bar{\pi }} }{d(\mu \otimes \nu )} = e^{\varphi + \psi - \varepsilon ^{-1} c_\lambda } \quad \mu \otimes \nu \text {-a.s.} $$
  for measurable functions $\varphi , \psi$ satisfying (11) and (12), then ${\bar{\pi }} = \pi _{\varepsilon }^*$.

Eq. (11) and (12) make explicit the link between the E-ROBOT and the Schrödringer equations, as in (4) and (5).

We notice that the expression for the potential can be rewritten in the equivalent exponential form. E.g. for (11), we have

$$\begin{aligned} \varphi ^*(x) &  =-\varepsilon \ln \int e^{\frac{\psi ^*(y)-c_\lambda (x, y)}{\varepsilon }} \nu (d y) \Longleftrightarrow e^{-\frac{\varphi ^*(x)}{\varepsilon }}\\  &  =\int e^{\frac{\psi ^*(y)-c_\lambda (x, y)}{\varepsilon }} \nu (d y). \end{aligned}$$

In our proofs, we make use of both representations. Moreover, since $c_\lambda \in L^{1}(\mu \otimes \nu )$, we state

#### Proposition 2

(E-ROBOT Dual problem) The $C_\epsilon$ in (9) and the related E-ROBOT in (10) are such that:

13 $$\begin{aligned} \inf _{\pi \in \Pi (\mu , \nu )} C_\varepsilon &  = \sup _{\varphi \in L^1(\mu ), \psi \in L^1(\nu )} \left\{ \int \varphi \, d\mu + \int \psi \, d\nu \right. \nonumber \\  &  \quad \left. - \varepsilon \int e^{\varphi + \psi - \varepsilon ^{-1} c_\lambda } \, d(\mu \otimes \nu ) + \varepsilon \right\} , \end{aligned}$$

and the supremum is attained by the E-ROBOT potentials $\varphi ^*, \psi ^* \in L^1(\mu ) \times L^1(\nu )$, with

14 $$\begin{aligned} \inf _{\pi \in \Pi (\mu , \nu )} C_\varepsilon = \varepsilon \left( \int \varphi ^* \, d\mu + \int \psi ^* \, d\nu \right) . \end{aligned}$$

The maximizers are a.s. unique up to an additive constant.

In the next section, equipped with the results of Propositions 1 and 2, we now study the main properties of the E-ROBOT problem and of some of its related notions.

## Main results: key properties of E-ROBOT

### Potentials: uniform boundedness and uniform convergence

The solution to the SBP described in Eq. (4) and (5) illustrates the pivotal role played by Schrödinger potentials. Propositions 1 and 2 further demonstrate that these potentials are fundamental to the solution of the E-ROBOT primal and dual problem. This leads to natural questions regarding the limiting behavior of the potentials in (11) and (12), as the regularization parameter approaches zero or as the sample size diverges. The next two propositions answer these questions. Specifically, Proposition 3 shows that the potentials are Lipschitz continuous and uniformly bounded functions.

#### Proposition 3

Let $X,Y \subset \mathbb {R}^d$ and $c_\lambda : X \times Y \rightarrow \mathbb {R}$ be the continuous and bounded ROBOT cost function. Let $\mu \in \mathcal {P}(X), \nu \in \mathcal {P}(Y)$ be probability measures, and let $\varphi ^*, \psi ^*$ be the optimal dual potentials for the entropic optimal transport problem with cost $c_\lambda$ and regularization parameter ε>0. Then $\varphi ^*$ and $\psi ^*$: (i) are Lipschitz continuous functions on X and Y, respectively; (ii) $\varphi ^* \in L^\infty (X)$ and $\psi ^* \in L^\infty (Y)$, namely they are uniformly bounded.

Besides being uniformly bounded, in the next proposition, we show also that the potentials converge uniformly as the sample size *n* diverges.

#### Proposition 4

Let $\mu _n, \nu _n$ be empirical measures based on *n* i.i.d. samples from compactly supported probability measures $\mu , \nu$ on compact sets $X,Y \subset \mathbb {R}^d$. Let $\phi ^*_{n}, \psi ^*_{n}$ be the Schrödinger potentials associated with the entropic optimal transport plan $\pi _n^* \in \Pi (\mu _n, \nu _n)$, and $\phi ^*, \psi ^*$ be the potentials associated with the true plan $\pi ^* \in \Pi (\mu , \nu )$, both for cost $c_\lambda$ and regularization parameter ε>0. Let $c_\lambda$ be the ROBOT cost function. Then, as $n \rightarrow \infty$,

$$ \sup _{x \in X} |\phi ^*_{n}(x) - \phi ^*(x)| \rightarrow 0, \quad \sup _{y \in Y} |\psi ^*_{n}(y) - \psi ^*(y)| \rightarrow 0 $$

i.e., the potentials converge uniformly.

A comment is in order. As noticed by an anonymous referee, an alternative proof of Proposition 4 can be obtained removing Proposition 3 and applying directly the arguments in Proposition 3.2 of Nutz and Wiesel (2022). Indeed, under the hypotheses of Proposition 4, one can deduce the uniform boundedness and equicontinuity of the Schrödinger potentials directly from their Proposition 3.2, and then apply Arzelà–Ascoli theorem. However, we prefer to keep Proposition 3 because it is formulated without compactness assumptions and exploits the specific structure of the truncated ROBOT cost to obtain global Lipschitz and boundedness properties of the potentials on arbitrary $X,Y\subset \mathbb {R}^d$. We remark that this provides an additional insight about E-ROBOT. We refer to the Supplementary Material for further detail.

### Optimal regularized transport plan

It is easy to conjecture that the structure of the E-ROBOT potentials and their limiting behavior have implications on the limiting behavior of the related transportation plan. In this section, we study that aspect. More precisely, for fixed λ, we discuss the limiting behavior as $\varepsilon \rightarrow 0$ of the E-ROBOT problem. The optimal transport plan $\pi _0$ of the corresponding unregularized OT problem is defined via:

15 $$\begin{aligned} C_0 = \inf _{\pi \in \Pi (\mu ,\nu )} \int c_\lambda \, d\pi . \end{aligned}$$

Since the value function $\inf _{\pi \in \Pi (\mu , \nu )} C_\varepsilon (\pi )$ of E-ROBOT decreases monotonically as $\varepsilon \downarrow 0$, we have

$$ \lim _{\varepsilon \rightarrow 0} \inf _{\pi \in \Pi (\mu , \nu )} C_\varepsilon (\pi ) \ge \inf _{\pi \in \Pi (\mu , \nu )} C_0(\pi ). $$

We now show this inequality is an equality, meaning the E-ROBOT value converges to the ROBOT value as regularization vanishes (Proposition 5(i)). Moreover, the corresponding optimizer converges weakly: the optimal plan $\pi ^*_\varepsilon$ for E-ROBOT converges to an optimal plan $\pi ^*_0$ for the original ROBOT problem with cost $c_\lambda$ (Proposition 5(ii)).

#### Proposition 5

Let $c_\lambda$ be the cost associated to the ROBOT problem for two measures μ and ν, and let $\pi _0$ be the optimal transport plan. If $H(\pi \Vert \mu \otimes \nu ) < \infty$, then:

(i) we have that
  16 $$\begin{aligned} \lim _{\varepsilon \rightarrow 0} \inf _{\pi \in \Pi (\mu , \nu )} C_\varepsilon = C_0. \end{aligned}$$
(ii) if $\varepsilon _n \rightarrow 0$ and $\lim _{n \rightarrow \infty } \pi ^{*}_{\varepsilon _n} = \pi _0$ weakly, we have that $\pi _0 \in \Pi (\mu ,\nu )$ is the unique unconstrained ROBOT plan and it follows that $\lim _{\varepsilon \rightarrow 0} \pi ^{*}_{\varepsilon } = \pi _0$ weakly.

Finally, we study the large sample behaviour ($n \rightarrow \infty$) of the regularized optimal transport plan. To this end, we state:

#### Proposition 6

Let $X,Y \subset \mathbb {R}^d$ be compact and $\mu _n \in \mathcal {P}(X), \nu _n \in \mathcal {P}(Y)$ be empirical measures based on i.i.d. samples from compactly supported probability measures $\mu , \nu$, such that $H(\pi \Vert \mu \otimes \nu ) < \infty$. Let $\pi _n^* \in \Pi (\mu _n, \nu _n)$ be the entropic optimal transport plan with cost $c_\lambda$ and regularization parameter ε>0, and let $\pi ^* \in \Pi (\mu , \nu )$ be the corresponding optimal plan for the true marginals. Then $\pi _n^* \rightarrow \pi ^*$ weakly as $n \rightarrow \infty .$

### Robust Sinkhorn divergence and MMD

Given the cost function $c_\lambda (x, y)$ defined on X×Y, with Y=X, and a regularization parameter ε>0, the entropic regularized optimal transport cost between two probability measures μ and ν is denoted $W_{\varepsilon ,\lambda }(\mu , \nu )$ and is defined as:

$$ W_{\varepsilon ,\lambda }(\mu , \nu ) = \inf _\pi \int c_\lambda \, d\pi + \varepsilon H(\pi \Vert \mu \otimes \nu ). $$

Due to the regularization, $W_{\varepsilon ,\lambda }(\mu , \mu )$ is not granted to be zero and this entails the entropic bias. To correct for this bias and ensure that the loss vanishes when $\mu = \nu$, we propose the following modified unbiased version:

17 $$\begin{aligned} \overline{W}_{\varepsilon ,\lambda }(\mu , \nu ) := W_{\varepsilon ,\lambda }(\mu , \nu ) - \frac{1}{2} \left( W_{\varepsilon ,\lambda }(\mu , \mu ) + W_{\varepsilon ,\lambda }(\nu , \nu ) \right) , \end{aligned}$$

and we call it the robust Sinkhorn loss. When $\lambda \rightarrow \infty$, $c_\lambda \rightarrow c$ so we obtain the usual Sinkhorn loss, which is commonly referred to as the Sinkhorn divergence in the OT literature that has been studied in Genevay et al. (2018, 2019).

The following proposition states that the robust Sinkhorn divergence $\overline{W}_{\varepsilon ,\lambda }$ defines a symmetric and positive definite loss function that is convex in each of its input variables. Moreover, it metrizes the convergence in law.

#### Proposition 7

Let $X \subset \mathbb {R}^d$ and consider the cost function $c_\lambda (x,y)$. Then, for all probability measures μ and ν on *X*, $\overline{W}_{\varepsilon ,\lambda }$ is such that:

(i) $$0= \overline{W}_{\varepsilon ,\lambda } (\nu , \nu ) \le \overline{W}_{\varepsilon ,\lambda } (\mu , \nu ), $$
(ii) $$\mu = \nu \iff \overline{W}_{\varepsilon ,\lambda } ( \mu , \nu ) = 0, $$
(iii) $$\lim _{n \rightarrow \infty } {\mu _n}= \mu \ \text {weakly} \ \iff \overline{W}_{\varepsilon ,\lambda } (\mu _n, \mu ) \rightarrow 0 $$.
(iv) (Limiting behavior for $\varepsilon \rightarrow 0$) $\overline{W}_{\varepsilon ,\lambda }(\mu , \nu ) \rightarrow W_\lambda (\mu , \nu ),$ as $\varepsilon \rightarrow 0.$

In addition to the limiting behavior for $\varepsilon \rightarrow 0$ discussed in Proposition 7, $\overline{W}_{\varepsilon ,\lambda }$ has connections to MMD (see e.g. Gretton et al. (2006)) and Bregman-type divergence (see e.g. Pardo (2018) for book-length introduction). Gretton et al. (2006) introduced MMD in machine learning and since then they have been applied for different tasks, like e.g. comparing distributions via distribution-free tests Gretton et al. (2012), generative models Li et al. (2015), gradient flow and neural network optimization Arbel et al. (2019). To illustrate the connection, let us consider

18 $$\begin{aligned} \Vert \mu - \nu \Vert ^2_{- c_\lambda } := \iint -c_{\lambda }(x,y) \, d(\mu -\nu )(x) \, d(\mu -\nu )(y), \end{aligned}$$

which is the MMD with kernel $- c_\lambda$. We refer to Lemma 12 in Appendix A for the properties of this kernel, here we remark that expanding the last expression, we obtain

19 $$\begin{aligned} \Vert \mu - \nu \Vert ^2_{- c_\lambda } &  = \iint - c_\lambda \, d\mu (x)\, d\mu (y) \nonumber \\  &  \quad \ + \iint - c_\lambda \, d\nu (x)\, d\nu (y) \nonumber \\  &  \quad \ - 2 \iint - c_\lambda \, d\mu (x)\, d\nu (y). \end{aligned}$$

From the definition of $W_{\lambda ,\varepsilon }(\mu , \nu )$, when $\varepsilon \rightarrow +\infty$, the entropic term $\varepsilon \, H(\pi \,\Vert \, \mu \otimes \nu )$ dominates. The solution to the E-ROBOT problem is then close to the independent coupling and the transport cost term becomes $\iint c_\lambda (x,y) \, d\mu (x) \, d\nu (y).$ So, as $\varepsilon \rightarrow \infty$, for each $W_{\lambda ,\varepsilon }$ in (17) we have

$$ \begin{aligned} W_{\lambda ,\varepsilon }(\mu , \nu )&\rightarrow \iint c_\lambda (x,y)\, d\mu (x) d\nu (y),\\ W_{\lambda ,\varepsilon }(\mu , \mu )&\rightarrow \iint c_\lambda (x,y)\, d\mu (x) d\mu (y),\\ W_{\lambda ,\varepsilon }(\nu , \nu )&\rightarrow \iint c_\lambda (x,y)\, d\nu (x) d\nu (y). \end{aligned} $$

Thus, as $\varepsilon \rightarrow \infty$ we have

20 $$\begin{aligned} \overline{W}_{\varepsilon ,\lambda } (\mu ,\nu ) &  \rightarrow \iint c_\lambda (x,y)\, d\mu (x) d\nu (y)\nonumber \\  &  \quad - \frac{1}{2} \left( \iint c_\lambda (x,y)\, d\mu (x) d\mu (y) \right. \nonumber \\  &  \quad \left. + \iint c_\lambda (x,y)\, d\nu (x) d\nu (y) \right) , \end{aligned}$$

and, comparing (20) to (19), we conclude that

21 $$\begin{aligned} \lim _{\varepsilon \rightarrow \infty } \overline{W}_{\varepsilon ,\lambda } (\mu ,\nu ) = \frac{1}{2} \Vert \mu - \nu \Vert ^2_{- c_\lambda } \end{aligned}$$

namely, in the large-ε limit, $\overline{W}_{\varepsilon ,\lambda } (\mu ,\nu )$ becomes half the squared MMD norm with kernel $- c_\lambda$. This results provide an interesting interpretation for the E-ROBOT: by changing ε, the E-ROBOT interpolates between the ROBOT and the MMD norm as obtained using the truncated cost as kernel.

In addition to this property, making use of the positive and *c*-universal kernel $k_{\varepsilon ,\lambda }$ (see Lemma 12 in Appendix A), we define the MMD:

22 $$\begin{aligned} \Vert \mu \Vert ^2_{k_{\varepsilon ,\lambda }} &  = \iint k_{\varepsilon ,\lambda } d\mu (x) d\mu (y)\nonumber \\  &  = \iint e^{- \frac{1}{\varepsilon } c_\lambda (x,y)} \, d\mu (x) d\mu (y), \end{aligned}$$

and we define also the E-ROBOT negentropy $F_{\varepsilon , \lambda }(\mu )$ as a mapping from $\mathcal {P}(X)$ to $\mathbb {R}$:

23 $$\begin{aligned} F_{\varepsilon , \lambda }: \mu \mapsto - \frac{1}{2} W_{\varepsilon ,\lambda }(\mu , \mu ) = - \frac{1}{2} \inf _{\pi \in \Pi (\mu , \mu )} C_\varepsilon (\mu , \mu , c_\lambda , \pi ) \end{aligned}$$

Working along the same lines as Proposition 4 in Feydy et al. (2019) (see Appendix A, Lemma 2), we can prove that

24 $$\begin{aligned} \frac{1}{\varepsilon } F_{\varepsilon , \lambda }(\mu ) + \frac{1}{2} = \inf _{\xi \in \mathcal {P}(X)} \left\{ \int \ln \left( \frac{d\mu }{d\xi } \right) d\mu + \frac{1}{2} \Vert \mu \Vert ^2_{k_{\varepsilon ,\lambda }} \right\} , \end{aligned}$$

where $F_{\varepsilon , \lambda }$ is a strictly convex functional on $\mathcal {P} (X)$. Moreover, $F_{\varepsilon , \lambda }$ is differentiable in the following sense.

Let $\mathcal {C}(X)$ denote the set of continuous function on *X*, and define the operator $\langle \cdot , \cdot \rangle : \mathcal {P}(X) \times \mathcal {C}(X) \rightarrow \mathbb {R}$ as the mapping $(\mu , f) \mapsto \int f(x) d\mu (x)$. Recall that a function $F: \mathcal {P}(X) \rightarrow \mathbb {R}$ is differentiable at $\mu \in \mathcal {P}(X)$ if there exists a continuous function (called gradient) $\nabla F(\mu ) \in \mathcal {C}(X)$ such that for any $\xi = \nu _1 - \nu _2$ with $\nu _1, \nu _2 \in \mathcal {P}(X)$, we have

$$F(\mu + t\xi ) = F(\mu ) + t \langle \xi , \nabla F(\mu ) \rangle + o(t).$$

Then, moving along the same lines as the proof in Appendix B2 of Feydy et al. (2019), we have the following proposition, which implies that the E-ROBOT negentropy $F_{\varepsilon , \lambda }$ is differentiable everywhere in $\mathcal {P} (X)$ with gradient $\nabla F_{\varepsilon , \lambda }(\mu )(x) = -\varphi ^*(x)/2$ for x∈X.

#### Proposition 8

Let $\mu , \nu \in \mathcal {P}(X)$ be probability measures on a compact set $X \subset \mathbb {R}^d$, and let $c_\lambda : X \times X \rightarrow \mathbb {R}$ be the ROBOT cost function. Then the entropic cost $W_{\varepsilon ,\lambda }(\mu , \nu )$ is weak-* continuous and differentiable over $\mathcal {P}(X) \times \mathcal {P}(X)$, with the gradient given by the pair of Schrödinger potentials:

$$ \nabla W_{\varepsilon ,\lambda }(\mu , \nu ) = (\varphi ^*, \psi ^*). $$

In Section 4.3, we show that the formula of the gradient $\nabla F_{\varepsilon , \lambda }(\mu )$ can be applied to model the evolution of a distribution along the E-ROBOT gradient flow. Also, it allows us to define the E-ROBOT Hausdorff divergence

25 $$\begin{aligned} H_{\varepsilon , \lambda }(\mu , \nu ) := \frac{1}{2} \left\langle \mu - \nu , \nabla F_{\varepsilon , \lambda }(\mu ) - \nabla F_{\varepsilon , \lambda }(\nu ) \right\rangle , \end{aligned}$$

which is a Bregman-type divergence induced by the strictly convex functional $F_{\varepsilon , \lambda }$ and is therefore a positive definite quantity.

### Sample complexity

The results of §3.3 illustrate the properties of $\overline{W}_{\varepsilon ,\lambda }$ as a tool to measure the proximity between two (probability) measures. This is similar to $W_p$, $W^{\lambda }$, and $W_{\varepsilon }$. However, the results of §3.3 are stated at the population level. In applications, $W_p$ and $W^{\lambda }$ are estimated from samples. A well-known issue is that the error of these empirical estimates suffers from a serious dependence on dimension: the rate at which $W_p(\hat{\mu }_n, \mu )$ and $W^{(\lambda )}(\hat{\mu }_n, \mu )$ converge to 0 scales as $n^{-1/d}$ under mild moment conditions for d≥3. Thus, this rate (also called sample complexity) deteriorates poorly with dimension. As shown in Genevay et al. (2019), $W_{\varepsilon }$ does not suffer from the same problem: its sample complexity scales with $n^{-1/2}$ in any dimension *d*. In Theorem 9 and Corollary 10, we show that $\overline{W}_{\varepsilon ,\lambda }$ enjoys the same desirable property.

Before presenting the statements, we clarify an important theoretical point. Although our results on the sample complexity of $\overline{W}_{\varepsilon ,\lambda }$ are consistent with those obtained for the Sinkhorn divergence in E-OT by Genevay et al. (2019) and for Gaussian-smoothed OT in Nietert et al. (2021), our proof requires a fundamentally different derivation and cannot directly build upon existing strategies. For instance, the proofs in Genevay et al. (2019) require that the cost function *c* is smooth, a condition not satisfied by our truncated cost $c_\lambda$. The proofs in Nietert et al. (2021) hinge on the specific idea of convolving measures with an isotropic Gaussian density, which is not a feature of our $\overline{W}_{\varepsilon ,\lambda }$ framework. Therefore, to establish the sample complexity of $\overline{W}_{\varepsilon ,\lambda }$, we must resort to different mathematical tools (empirical process theory), leveraging the special structure of the E-ROBOT potentials.

The novelty of our approach lies in this application of empirical process theory to the E-ROBOT potentials, whose regularity properties are guaranteed by the entropic regularization. To provide intuition for this strategy, recall that $\overline{W}_{\varepsilon ,\lambda }(\mu ,\nu )$ depends on ${W}_{\varepsilon ,\lambda }(\mu ,\nu )$, which in turn depends on the entropic regularization term $\varepsilon H(\pi \Vert \mu \otimes \nu )$. Now, recall that

$$ H(\pi \Vert \mu \otimes \nu ) = \int \ln \left( \frac{d\pi }{d(\mu \otimes \nu )} \right) d\pi , $$

where the optimal plan $\pi ^*$ is:

$$ \frac{d\pi ^*}{d(\mu \otimes \nu )}(x,y) = \exp \left( \varphi ^*(x) + \psi ^*(y) - \frac{1}{\varepsilon } c_\lambda (x,y) \right) , $$

and similarly for $\pi _n^*$ with potentials $\varphi _n^*, \psi _n^*$ and empirical marginals $\mu _n, \nu _n$. Substituting into the entropy expression, we obtain:

$$ H(\pi ^* \Vert \mu \otimes \nu ) = \int \left( \varphi ^*(x) + \psi ^*(y) - \frac{1}{\varepsilon } c_\lambda (x,y) \right) d\pi ^*(x,y). $$

Now, since $\varphi _n^*, \psi _n^* \rightarrow \varphi ^*, \psi ^*$ uniformly (see Proposition 4), and $c_\lambda$ is bounded and Lipschitz, the integrand $(x,y) \mapsto \varphi _n^*(x) + \psi _n^*(y) - c_\lambda (x,y)/{\varepsilon }$ is itself uniformly bounded and Lipschitz. Moreover, from Proposition 5, the plans $\pi _n^* \rightarrow \pi ^*$ weakly, and the domain is compact. By standard empirical process theory (uniform convergence for Lipschitz function classes, see Sections 2.2 and 2.5.1 in Van Der Vaart and Wellner (1996)), we obtain $\mathbb {E} \bigg [ \bigg | H(\pi _n^* \Vert \mu _n \otimes \nu _n) - H(\pi ^* \Vert \mu \otimes \nu ) \bigg | \bigg ] = \mathcal {O}(n^{-1/2})$. This follows because the Schrödinger potentials $\varphi _n^*, \psi _n^*$ are uniformly bounded and Lipschitz (see Proposition 3), and their uniform convergence (see Proposition 4) ensures that the integrand in the entropy expression is itself Lipschitz and bounded. Therefore, $H(\pi _n^* \Vert \mu _n \otimes \nu _n) - H(\pi ^* \Vert \mu \otimes \nu )$ behaves like an empirical process indexed by a class of functions that is both Glivenko–Cantelli and Donsker, and its expectation converges at the rate $n^{-1/2}$.

The above derivation provides the basic intuition for how the SBP, the ROBOT, and the empirical process theory can be combined nicely to establish the sample complexity of $\overline{W}_{\varepsilon ,\lambda }(\mu ,\nu )$. With this regard, we state:

#### Theorem 9

Let $\mu _n, \nu _n$ be empirical measures based on *n* i.i.d. samples from compactly supported probability measures $\mu , \nu$ on compact set $X \subset \mathbb {R}^d$. Let $c_\lambda$ be a bounded Lipschitz cost function and ε>0 a fixed regularization parameter. Then the expected deviation of the robust Sinkhorn loss satisfies:

$$ \mathbb {E}\left[ \left| \overline{W}_{\varepsilon ,\lambda }(\mu _n, \nu _n) - \overline{W}_{\varepsilon ,\lambda }(\mu , \nu ) \right| \right] = \mathcal {O}(n^{-1/2}). $$

#### Corollary 10

Under the same assumptions as Theorem 9, we have $\mathbb {E}\left[ \overline{W}_{\varepsilon ,\lambda }(\mu _n, \mu ) \right] = \mathcal {O}(n^{-1/2}).$

#### Remark 11

The proofs of these results reveal an important aspect. A key distinction between E-ROBOT and ROBOT lies in the structure of their respective dual potentials. In E-ROBOT, the Schrödinger potentials $\phi ^*, \psi ^*$ satisfy fixed-point equations in (4) and (5), which correspond to a SoftMax operation; see Peyré and Cuturi (2019), Ch. 4. This structure induces smoothness and regularity, ensuring that the potentials are uniformly bounded and Lipschitz continuous. As a result, the function class indexed by these potentials has finite entropy and is P-Donsker under compact support. In contrast, the ROBOT framework lacks entropic regularization, and its Kantorovich potentials arise from a linear program with Lipschitz and range constraints. While these potentials are bounded and Lipschitz, they do not enjoy the smoothing effects of the SoftMax structure. Consequently, uniform convergence and Donsker-type properties are not guaranteed in ROBOT when d≥2.

### Truncated Laplace deconvolution

Rigollet and Weed (2018) give a statistical interpretation of E-OT for $W_2$ by showing that performing maximum-likelihood estimation for Gaussian deconvolution corresponds to calculating a projection with respect to the entropic optimal transport distance. The projection estimator as obtained using $W_2$ has been employed in the machine learning community as a smoothed version of a minimum Kantorovich distance estimator (MKE, Bassetti et al. (2006); Bassetti and Regazzini (2006)) more suitable for automatic differentiation in generative models; see Montavon et al. (2016), Genevay et al. (2018, 2019). We now prove that this connection between E-OT and maximum-likelihood deconvolution can be derived also for the E-ROBOT, considering the deconvolution with a truncated Laplace distribution.

To elaborate, we start by recalling that a class $\mathcal {P}$ of probability measures is said to be closed under domination, if $\mu _1 \ll \mu _2$ for some $\mu _2 \in \mathcal {P}$ implies that $\mu _1 \in \mathcal {P}$. Moreover, let $\mathcal {\mu }$ contain probability distributions over $\mathbb {R}^d$ and let $\mu ^*$ be an unknown distribution of an i.i.d. sample $X_1, \ldots , X_n$. The deconvolution problems consists in estimating $\mu ^*$ using the corrupted random observations $(Q_1, \ldots , Q_n)$, where

26 $$\begin{aligned} Q_i=X_i+Z_i, \quad i=1, \ldots , n \end{aligned}$$

and the errors $Z_1, \ldots , Z_n$ are independent of $X_1, \ldots , X_n$. In what follows, the random variables $\left\{ Z_i\right\}$ are assumed to be independent copies of a random variable *Z* with known truncated Laplace distribution: $Z \sim \mathcal {L}\left( 0, \varepsilon , \lambda \right)$, where the location is zero, the scale is ε, and the truncation parameter is λ.

In this context, the distribution of $Q_i$ has density $f_{\left( 0, \varepsilon , \lambda \right) } \star \textrm{d} \mu ^*$, where, for any $\mu \in \mathcal {P}$, we define

$$ f_{\left( 0, \varepsilon , \lambda \right) } \star \textrm{d} \mu (y)=\int f_{\left( 0, \varepsilon , \lambda \right) } (y-x) \textrm{d} \mu (x) $$

and $f_{\left( 0, \varepsilon , \lambda \right) }$ denotes the density of $Z \sim \mathcal {L}\left( 0, \varepsilon , \lambda \right)$. Under these assumptions, we call (26) the truncated Laplacian deconvolution model. The maximum-likelihood estimator (MLE) $\hat{\mu }$ defined by

$$ \hat{\mu }=\underset{\mu \in \mathcal {P}}{\operatorname {argmax}} \sum _{i=1}^n \ln f_{\left( 0, \varepsilon , \lambda \right) } \star \textrm{d} \mu \left( Q_i\right) $$

is a natural candidate to estimate $\mu ^*$.

Equipped with these definitions, we state a proposition that makes the link between the E-ROBOT and the truncated Laplacian deconvolution model, showing that E-ROBOT is in fact implementing $\hat{\mu }$.

#### Proposition 12

Let $\nu _n = {\sum _{i=1}^n \delta _{q_i}}/{n}$ be an empirical measure of the observations $(q_1,q_2,\ldots ,q_n)$. Let $\mathcal {P}$ be a convex class of probability measures that is closed under domination. The maximum-likelihood estimator for the Laplace convolution model Q=X+Z, where the noise *Z* has truncated Laplace density $f_{\left( 0, \varepsilon , \lambda \right) } (z) \propto \exp (-\varepsilon ^{-1} c_\lambda (0, z))$, is given by

$$ \hat{\mu } = \arg \min _{\mu \in \mathcal {P}} W_{\lambda ,\varepsilon }(\mu , \nu _n). $$

Proposition 12 implies that the maximum-likelihood estimator $\hat{\mu }$ is the projection of the empirical measure $\nu _n$ onto $\mathcal {P}$ with respect to $W_{\lambda ,\varepsilon }$. Differently from the $W_2$ case of Rigollet and Weed (2018), the E-ROBOT framework corresponds to a deconvolution problem with a specific, robust noise model: the noise variable *Z* is assumed to follow a truncated Laplacian distribution. This distribution has the following properties: (i) for $\Vert z\Vert \le 2\lambda$, $f_{0,\varepsilon , \lambda }(z) \propto \exp (-\Vert z\Vert /\varepsilon )$, identical to a standard Laplace distribution; (ii) for $\Vert z\Vert > 2\lambda$, $f_{0,\varepsilon , \lambda }(z) \propto \exp (-2\lambda /\varepsilon )$, a constant value. This implies a uniform distribution on the tails beyond the radius 2λ; (iii) the log-probability of any large value is bounded below by $-{2\lambda }/{\varepsilon }$, making the model robust to outlying observations. The parameter λ controls the robustness (truncation point), while ε controls the scale (dispersion) of the core Laplace distribution component. Proposition 12 justifies the use of E-ROBOT for inference and prediction tasks, like those described in Ma et al. (2025)—e.g. minimum Kantorovich distance estimation, generative models, domain adaptation, and outliers detection.

## Numerical illustrations

We illustrate the benefits and ease-of-use of E-ROBOT with four examples, covering key statistical inference issues and typical machine learning problems. All calculations were performed on a standard laptop with a 2.4 GHz 8-Core Intel Core i9 processor, with each exercise requiring only a few minutes. Code to replicate our results is available on GitHub at https://github.com/dvdlvc/E-ROBOT and can be combined with the ROBOT code available at https://github.com/dvdlvc/Robust-optimal-transportation.

A methodological note on implementation. The application of E-ROBOT requires selecting the hyper-parameters λ (from ROBOT) and ε (from E-OT). A theoretically grounded, general-purpose procedure for this joint selection remains a fundamental open challenge—rather than a limitation specific to this work. The literature, in fact, offers scant guidance even on selecting these parameters in isolation, with some exceptions like the ROBOT of Ma et al. (2025). We regard the derivation of such a joint selection criterion as an essential but separate line of theoretical research—see §5. In practice, we emphasize that E-ROBOT is highly operable. We demonstrate that small values of ε, consistent with standard E-OT procedures (see e.g. Peyré and Cuturi (2019)), yield excellent results across all our experiments (e.g., the power curves in §4.1 and barycenter calculations in §4.2.2) even for large dimensions. Furthermore, inheriting the stability of its ROBOT predecessor, the method performs effectively across a wide range of λ values (we refer to Figure 4 for numerical insights into the sensitivity of the power curves to λ). As a general rule for implementation, we advise analysts to inspect the truncated cost matrix: the distribution of its entries provides immediate, empirical, insight into the scale of outlier-induced costs and a suitable range for λ.

### Nonparametric tests for equality of multivariate distributions

The $\overline{W}_{\lambda ,\epsilon }$ and/or the related MMD or divergences described in §3.3 can be applied to devise testing procedures aimed to determine whether given two distribution are the same. More generally, they can be used as a loss functions in various statistics machine learning tasks such as density estimation, domain adaptation, and generative models. Among these different uses, we consider the Goodness-of-Fit (GoF) test problem discussed in §2 of Hallin et al. (2021) uses the empirical Wasserstein distance between the empirical distribution $\hat{\mu }_n$ and a fully specified null distribution $\mu _0$. Their test statistic is based on $W_p^p(\hat{\mu }_n, \mu _0)$, with critical values determined via Monte Carlo simulation under the null. While this approach is fully nonparametric, it is sensitive to outliers and suffers from the curse of dimensionality. To address these limitations, we propose replacing the classical Wasserstein distance with the robust Sinkhorn distance $\overline{W}_{\varepsilon ,\lambda }$ from the E-ROBOT framework. Specifically, we define the test statistic as:

27 $$\begin{aligned} \overline{T}_n := \overline{W}_{\varepsilon ,\lambda }(\hat{\mu }_n, \mu _0). \end{aligned}$$

This modification inherits the robustness properties of ROBOT and the statistical regularity of entropic optimal transport, including dimension-independent convergence rates and uniform convergence of the associated Schrödinger potentials.

Under the conditions of Proposition 7, one can prove a consistency result analogous to Proposition 1 in Hallin et al. (2021): for any fixed alternative $\mu \ne \mu _0$, the test based on $\overline{T}_n$ rejects the null hypothesis with probability tending to one as $n \rightarrow \infty$: $\lim _{n \rightarrow \infty } \mathbb {P}(\overline{T}_n > c_n(\alpha )) = 1,$ where $c_n(\alpha )$ is the Monte Carlo critical value at level α. This follows from the convergence of $\overline{W}_{\varepsilon ,\lambda }(\hat{\mu }_n, \mu )$ to $\overline{W}_{\varepsilon ,\lambda }(\mu , \mu _0)$ and the fact that $\overline{W}_{\varepsilon ,\lambda }(\mu , \mu _0) > 0$ under the alternative.

To implement the test, we compute $$\overline{T}_n$$ as in (27) using an adaptation of the Sinkhorn algorithm (see e.g. Peyré and Cuturi (2019)) for the ROBOT setting, as in the pseudocode available in Algorithm 1. Then, we apply it to the mentioned GoF problem. Specifically, we test two null hypotheses: first, $$\mathcal {H}_0: \mu _0 = \mathcal {N}(0_d, I_d)$$ (a *d*-variate standard normal); second, $$\mathcal {H}_0: \mu _0 = {t}(0_d, I_d, 1)$$, namely a *d*-variate *t*-distribution with df=1, location $$0_d$$ (the *d*-dimensional vector of zeros), and scale d×d-matrix $$ I_d$$. To examine the role of dimensionality *d*, we study power curves for $$d \in \{2,10,15\}$$, sample size n=50, significance level 5%, and a sequence of local alternatives obtained by shifting the location parameter equally in each dimension. For comparison, we also include the test statistic based on the $$W_1$$ distance from Hallin et al. (2021). Note that in this setting, $$\overline{W}_{\lambda ,\varepsilon }$$ is well-defined for all considered distributions, whereas $$W_1$$ is well-defined for the Gaussian cases but not for the *t*-distributions (where the first moment does not exist).

**Algorithm 1:**
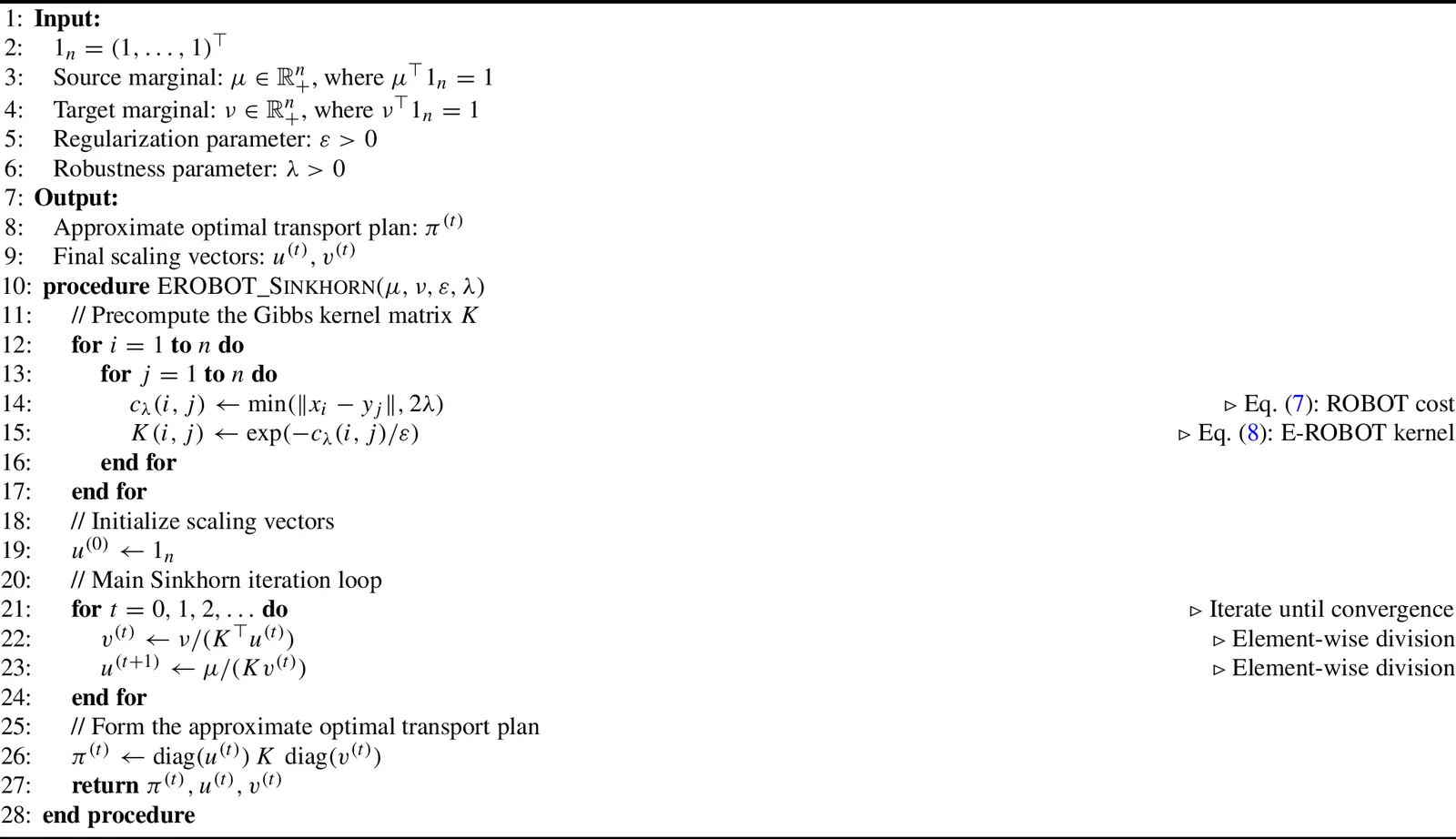
E-ROBOT Sinkhorn Algorithm

**Fig. 2:**
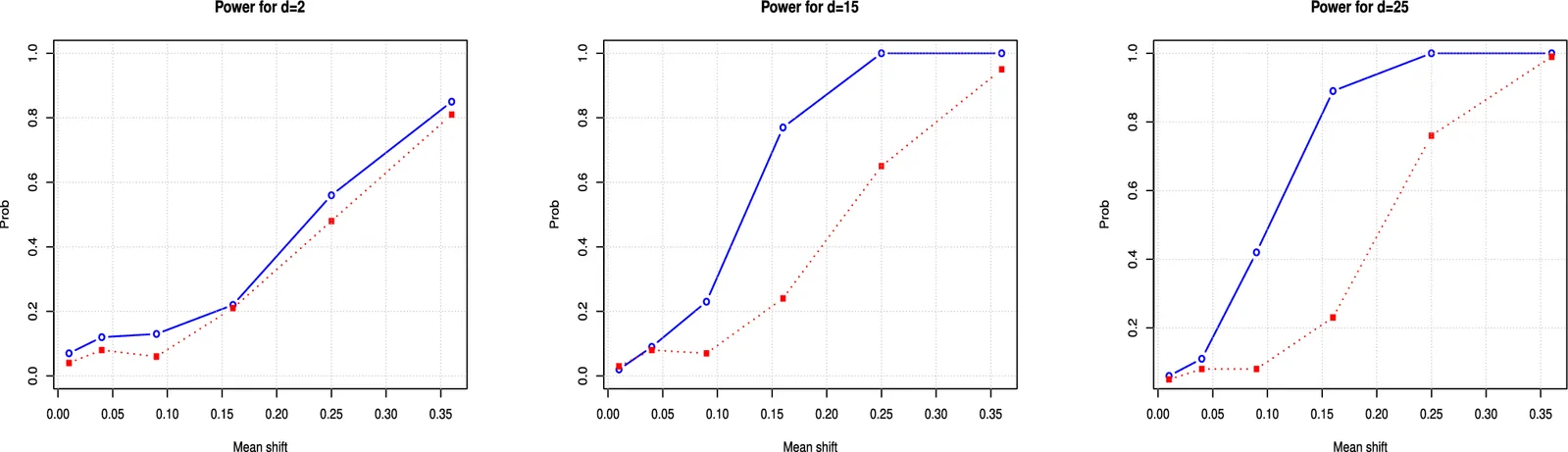
GoF power curves for testing the null of a *d*-variate standard normal. The continuous (blue) curve with circles corresponds to the $\overline{T}_n$ statistic using the entropic regularized Sinkhorn distance with ε=5 and λ=10; the dotted (red) curve with squares corresponds to the test based on $W_1$

Figure 2 presents the results for the multivariate normal case. The left plot indicates that for d=2, our test performs similarly to the $W_1$-based test. As the dimension increases, however, $\overline{T}_n$ begins to outperform its $W_1$-based counterpart. In the Supplementary Material, we complement the analysis and consider the t-distribution (as in Section 1.2): we illustrate that the $W_1$-based test operates near its nominal level, while $\overline{T}_n$ maintains strong power across all dimensions.

**Fig. 3:**
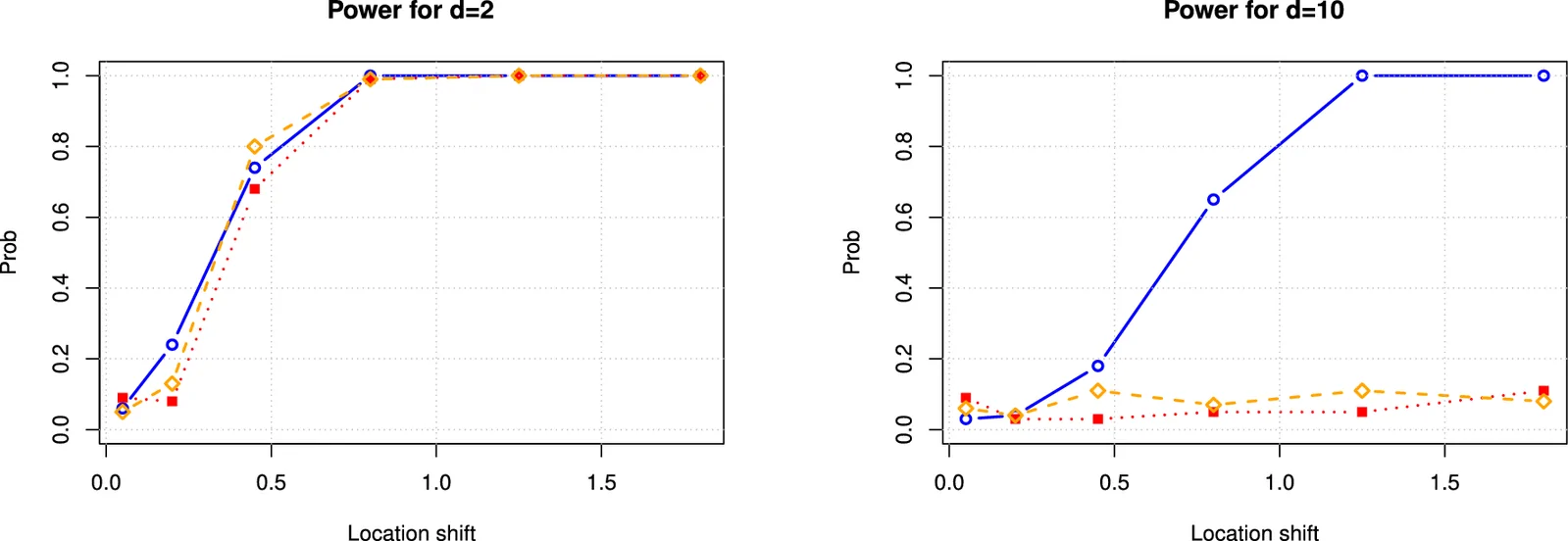
GoF power curves for testing the null of a *d*-variate *t*-distribution with d=2,df=4 (left panel), and d=10,df=1 (right panel), location zero, and identity scale matrix. The continuous (blue) curve with circles corresponds to the $\overline{T}_n$ statistic with ε=0.05 and λ=3; the dotted (red) curve with squares corresponds to the test based on $W_1$; the dashed (orange) curve with diamonds corresponds to the test based on Robust Mahalanobis Distance (RMD)

**Fig. 4:**
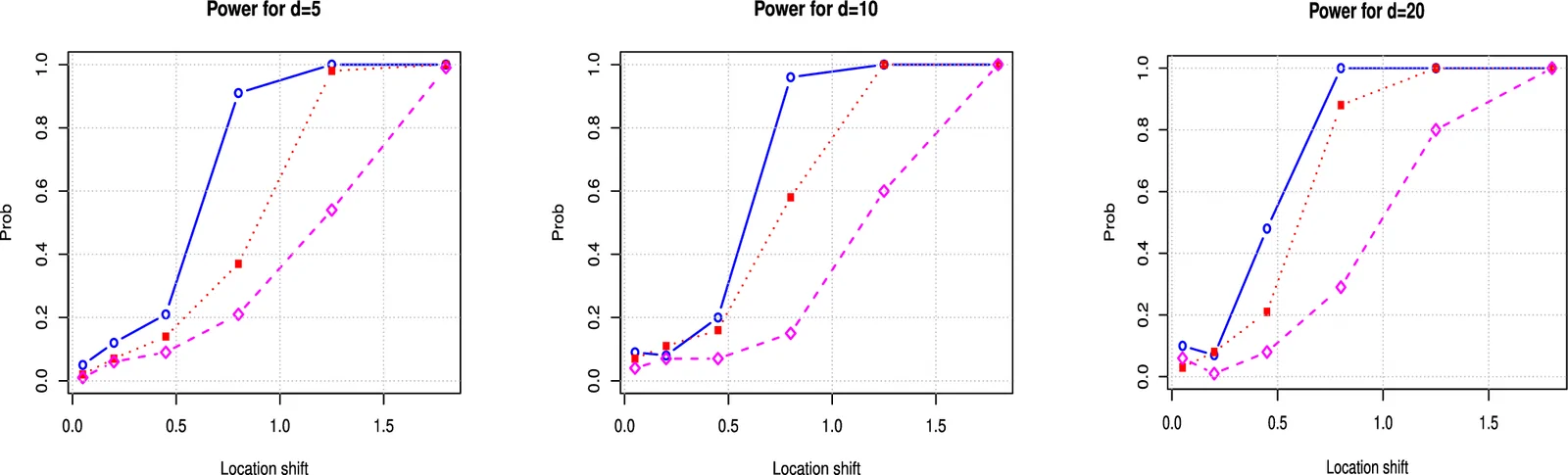
Sensitivity of the GoF power curves the choice of λ. We test the null of a *d*-variate *t*-distribution with different values of *d* and df=1, location zero, and identity scale matrix. The continuous (blue) curve with circles corresponds to the $\overline{T}_n$ statistic with λ=3; the dotted (red) curve with squares corresponds to the test based on $\overline{T}_n$ with λ=6; the dashed (magenta) curve with diamonds corresponds to the test based on $\overline{T}_n$ with λ=12. In all experiments we set ε=0.05

In the context of heavy-tailed t-distributions, we consider the robust Mahalanobis distance (RMD). For its numerical implementation, we use the routine available in the R package robustbase (default setting). The RMD is typically applied to elliptical distributions and, similarly to the E-ROBOT, it relies on reducing the weight of certain observations via trimming to estimate location and scale. We refer to Maronna et al. (2006, p. 190) for key methodological aspects, and to the R documentation for covMcd for computational details. Figure 3 displays the power curves for $\overline{T}_n$, the $W_1$-based test, and the RMD-based test. The plots illustrate that, when df=4 and d=2, all three tests perform similarly. However, as the dimension increases and the degrees of freedom decrease, both the $W_1$- and RMD-based tests suffer substantial power loss, whereas $\overline{T}_n$ retains its power. Finally, we conduct a sensitivity analysis to λ: Figure 4 displays power curves for various λ values and dimensions. showing that λ in the range [3,6] yields good power, which remains stable across all dimensions. Thus, we recommend using small values of ε and selecting λ in the interval [3,6]: it is reassuring to note that within this range, power differences across dimensions are moderate and the E-ROBOT test remains powerful across different choices of λ. A note of caution is warranted: these recommendations are specific to the settings examined here, and the broader problem of joint selection of λ and ε remains an open question for future research (see §5).

### E-ROBOT barycenters

#### IBP for E-ROBOT

Computing Wasserstein barycenters is a fundamental task in OT, with machine learning applications ranging from shape analysis and image synthesis to generative modeling. It provides a principled way to define a central representative of a collection of probability measures, generalizing the notion of the Fréchet mean to the Wasserstein space.

Let $\Sigma _n:= \left\{ \mu \in \mathbb {R}_+^n :\; \mu ^\top 1_n = 1 \right\}$ denote the simplex in $\mathbb {R}^n$. In the classical entropic barycenter formulation, the barycenter $\mu \in \Sigma _n$ of input measures $\mu _m \in \Sigma _n, m = 1, \ldots , M$, is defined as the solution to $\min _{\mu \in \Sigma _n} \sum _{m=1}^M \alpha _m W_\varepsilon (\mu _m, \mu )$. Given $(\mu , \nu ) \in \Sigma _n \times \Sigma _n$, the polytope of couplings between μ and ν is defined as

$$ \Pi (\mu , \nu ) := \left\{ \gamma \in \mathbb {R}_+^{n \times n} \;:\; \gamma {1}_n = \mu , \; \gamma ^\top {1}_n = \nu \right\} . $$

As expressed in (9), the E-OT problem can be re-cast in the form $\min _{\gamma \in \mathcal {S}} \text {KL}(\gamma \mid \xi )$ where ξ is a given point in $\mathbb {R}_{+}^{n \times n}$, and $\mathcal {S}$ is an intersection of two closed convex sets, namely $\mathcal {S}= \mathcal {S}_{1} \bigcap \mathcal {S}_{2}$, such that $\mathcal {S}$ has nonempty intersection with $\mathbb {R}_{+}^{n \times n}$, where $\mathcal {S}_1 = \left\{ \boldsymbol{\gamma }= (\gamma _m)_{m=1}^M \in (\Sigma _n )^M: \gamma ^\top _m {1}_n = \mu _m \quad \forall m \right\}$ and $\mathcal {S}_2 = \left\{ \boldsymbol{\gamma }= (\gamma _m)_{m=1}^M \in (\Sigma _n )^M : \exists \mu \in \mathbb {R}_+^{n}, \gamma _m {1}_n = \mu \ \forall m \right\}$. We focus on the case where the convex sets $\mathcal {S}_{\ell }$, for ℓ=1,2 are affine subspaces. In this case, it is possible to solve the KL -minimization problem by simply using iterative KL-projections. For the sake of completeness, we recall that, given a convex set *D* and a reference measure ξ, the KL-projection of ξ onto *D* is defined as

$$\begin{aligned} &  \textrm{P}_{D}^{\textrm{KL}}(\xi ) := \arg \min _{\gamma \in D} \\ &  \textrm{KL}(\gamma \Vert \xi ), \quad \textrm{KL}(\gamma \Vert \xi ) = \sum _{i,j} \gamma (i,j) \left( \ln \left( \frac{\gamma (i,j)}{\xi (i,j)} \right) - 1 \right) . \end{aligned}$$

To compute the KL-projection, we start from $\gamma ^{(0)}=\xi$, and compute ∀t>0, $\gamma ^{(t)}:= P_{\mathcal {S}_{t\text {mod}2}}^{\textrm{KL}}\left( \gamma ^{(t-1)}\right)$. One can show that $\gamma ^{(t)}$ converges towards the unique solution $\gamma ^{(t)} \rightarrow P_{\mathcal {S}}^{\textrm{KL}}(\xi )$ as $t \rightarrow \infty$; see Benamou et al. (2015).

The computation of the barycenter in the E-ROBOT setting, requires the computation of a Wasserstein barycenter of input probability measures $\mu _1, \dots , \mu _M \in \Sigma _n$ with weights $\alpha _1, \dots , \alpha _M$ (such that $\sum _m \alpha _m = 1$), using $W_{\lambda ,\varepsilon }$. To achieve this goal, we resort to KL-projections using the truncated cost matrix $C_\lambda \in \mathbb {R}^{N \times N}$ whose (*i*, *j*)-entry is $C_\lambda (i,j) = \min (C(i,j), 2\lambda )$, where $C({i,j})= d(x_i, y_j)$, and the associated Laplace kernel $\xi _k = \xi = k_{\lambda , \varepsilon }$ as in (8), for every m=1,2,…,M. Indeed, as in the E-OT case, the E-ROBOT barycenter problem can be re-formulated as:

28 $$\begin{aligned} \min _{ \boldsymbol{\gamma }} \sum _{m=1}^M \alpha _m \, \textrm{KL}(\gamma _m \Vert \xi _m), \quad \text {s.t.} \quad \boldsymbol{\gamma }\in \mathcal {S}_1 \cap \mathcal {S}_2. \end{aligned}$$

Then, the Iterative Bregman Projection (IBP) scheme (see Benamou et al. (2015)) can be applied. More specifically, we first compute the projection onto the constraint set $\mathcal {S}_1$ for each *k*, then we compute the projection onto the constraint set $\mathcal {S}_2$, which enforces a shared left marginal μ across all couplings. This procedures leads to iterates $\boldsymbol{\gamma }^{(t)} = (\gamma _{m}^{(t)})_{m}$ which satisfy, for each m, $\gamma _{m}^{(t)} = \textrm{diag}(u_{m}^{(t)}) \, \xi \, \textrm{diag}(v_{m}^{(t)})$ for two vectors $(u_{m}^{(t)}, v_{m}^{(t)}) \in \mathbb {R}^n \times \mathbb {R}^n$, initialized as $v_{m}^{(0)} = {1}_n$ for all m, and computed with the iterations:

$$ u_{m}^{(t)} = \frac{\mu ^{(t)}}{\xi v_m^{(t)}}, \quad v_m^{(t+1)} = \frac{\mu _m}{\xi ^\top u_m^{(t)}}, $$

where $\mu ^{(t)}$ is the current estimate of the barycenter obtained as: $\mu ^{(t)} = \prod _{m=1}^N \left( u_m^{(t)} \odot (\xi v_m^{(t)}) \right) ^{\alpha _m}.$ Operations are to be interpreted element-wise, namely, for vectors $(a, b) \in \mathbb {R}^n \times \mathbb {R}^n$, we denote entry-wise multiplication and division as

$$ a \odot b := (a_i b_i)_i \in \mathbb {R}^n \quad \text {and} \quad \frac{a}{b} := (a_i/b_i)_i \in \mathbb {R}^n. $$

These alternating projections are repeated until convergence: the resulting shared left marginal μ is the robust entropic barycenter. To summarize the described methodology, in Algorithm 2, we provide the pseudo code for E-ROBOT barycenters via IBP calculation.

**Algorithm 2:**
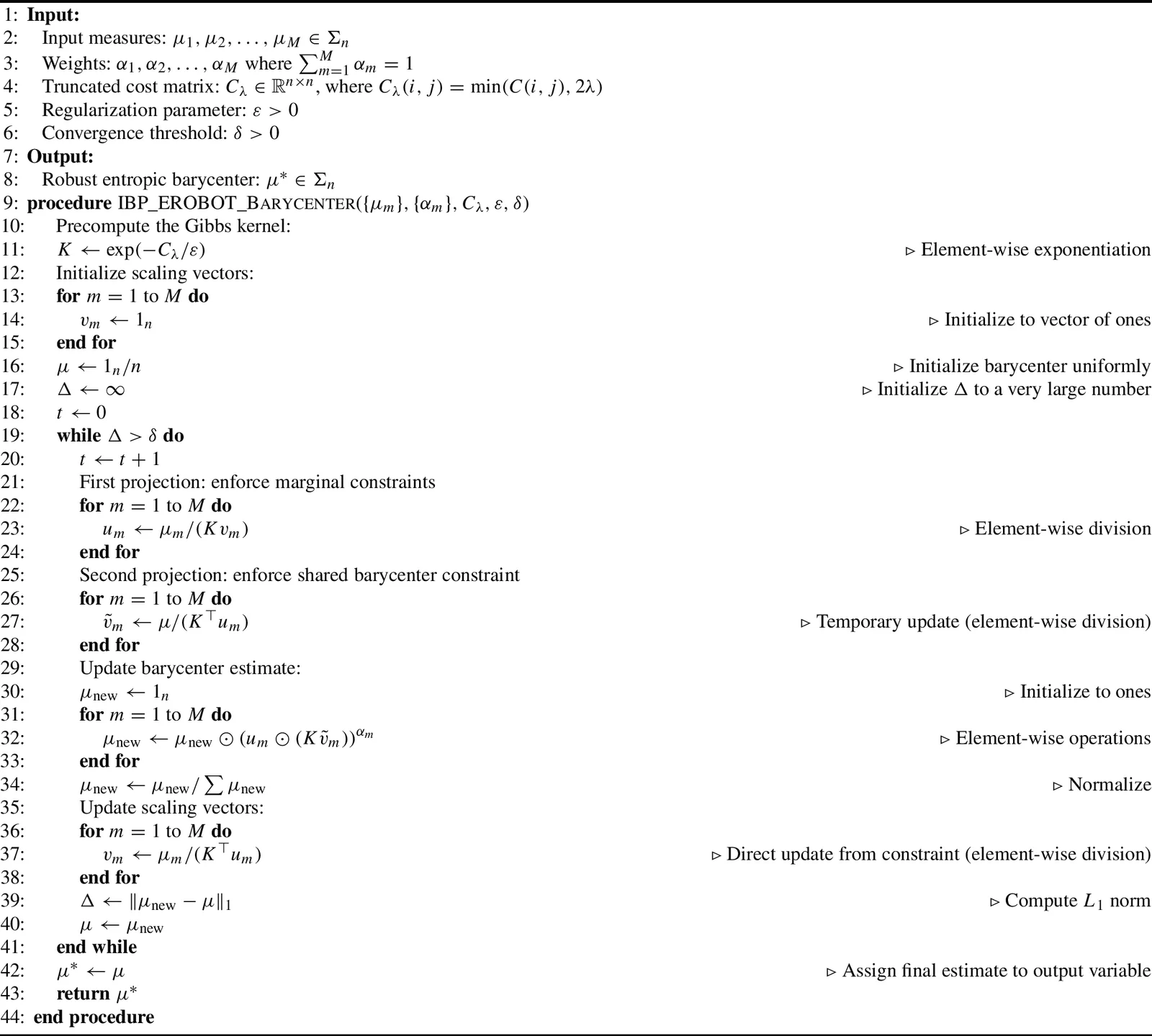
E-ROBOT Barycenter via Iterative Bregman Projections (IBP)

In the next two subsections, we illustrate how the E-ROBOT barycenters perform on 2D and 3D shapes in the presence of anomalous records. To implement our method, we resort to the Python library ot, which contains the routine bregman that computes Bregman projections for E-OT. That routine requires, as an input, a user-specified cost matrix. Therefore, to implement our E-ROBOT method we need to input in ot.bregman the matrix resulting from the application of the truncated cost function $c_\lambda$ to the 2D and 3D data, computing the distance via the routine cdist and trimming the entries of the resulting matrix via 2λ.

#### Barycenters for corrupted 2D and 3D shapes

In the next numerical example, we illustrate the use of the IBP and E-ROBOT in the computation of barycenters for 2D images. We consider two shapes. *Shape 1 (Source)* is a red circle with a radius of 4.5 pixels which we contaminate with 10 outliers in the top-right corner. *Shape 2 (Target)* is a blue square with a side length of 9 pixels which we contaminate with 10 outliers in the bottom-right corner. The resulting images are normalized to represent probability distributions.

**Fig. 5:**
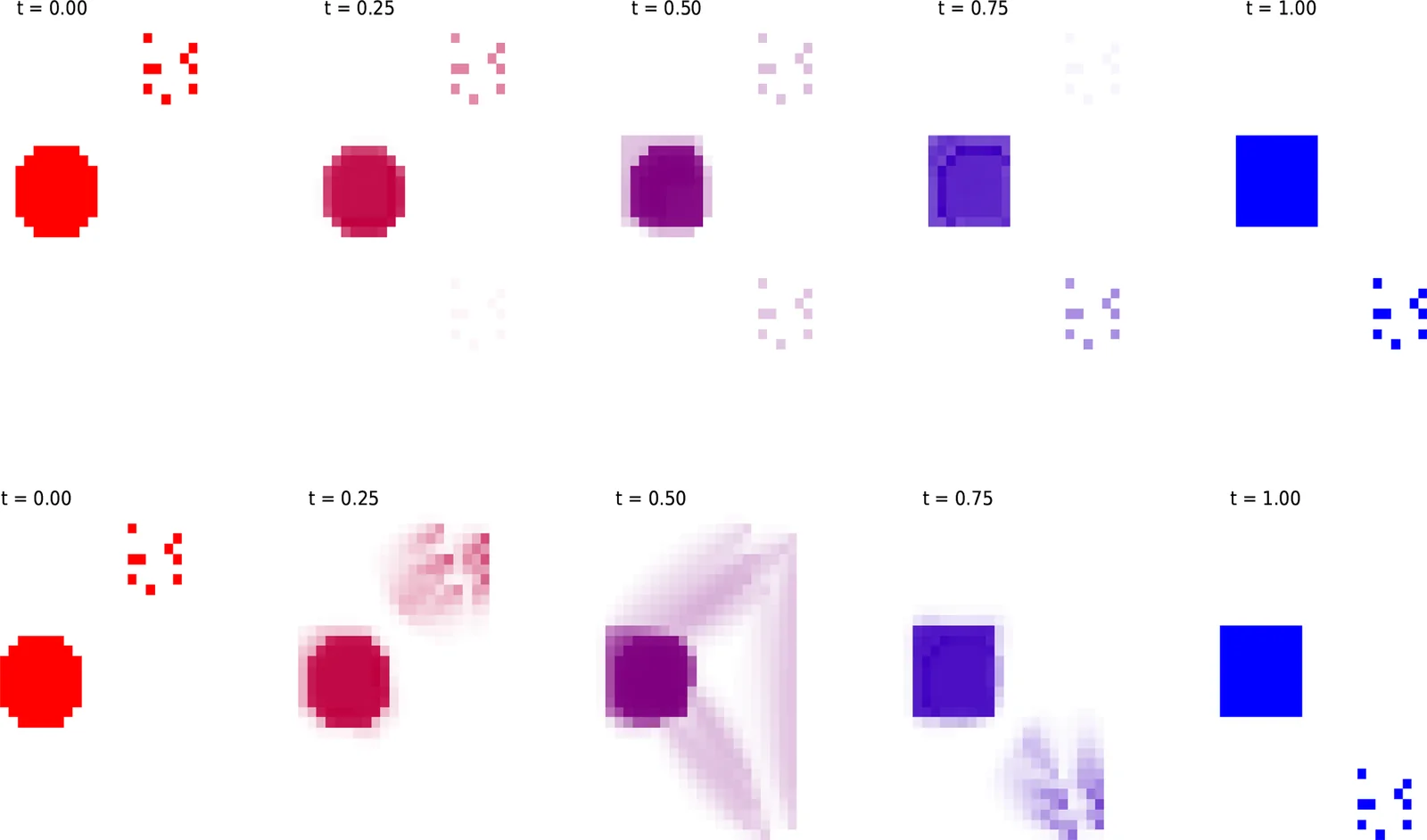
E-ROBOT Barycenters via IBP for 2D shapes: top panels for λ=4 and bottom for $\lambda =4 \times 10^7$. Shapes are on a 32×32 grid. The entropic regularization parameter is ε=0.15

We compute entropic barycenters, which can be interpreted as interpolated shapes between the source and target distributions. For the sake of visualization, we consider weights t=0.25,0.5,0.75, with corresponding the weights for the barycenter calculation being $\{1-t, t\}$. We consider the E-ROBOT and the E-OT. In the E-OT case, the cost matrix is based on the Euclidean distance between pixel coordinates as implemented in the Python routine entropic_barycenter in the ot library. To implement the E-ROBOT barycenters, we modify this routine introducing a truncation parameter λ, which trims the entires of this matrix. This simple modification is central to the implementation. Indeed, it allows for the comparison between two distinct methods: the standard E-OT and our novel E-ROBOT. In Figure 5, we display two sets of plots, one for each method.

The five top plots, corresponding to the E-ROBOT with a small λ=4, show a clean barycenter computation process: the red circle smoothly transforms into the blue square, while the outliers appear to simply fade in and out at their fixed locations without being transported. This demonstrates that E-ROBOT successfully isolates the barycenters computation of the main shapes from the influence of the outliers. Differently, the five bottom plots, which is for the E-ROBOT with a large λ, illustrate that the outliers significantly impact the barycenter calculation: as the circle becomes the square, the red outliers from the top-right corner are transported to the location of the blue outliers in the bottom-right corner. This results in particularly noticeable at t=0.5, where a blurred mass appears to be streaking between the outlier locations. This is due to the fact that the E-ROBOT with a large λ behaves like the standard E-OT approach: it attempts to transport all mass, including the anomalous records, as part of the overall transformation.

**Fig. 6:**
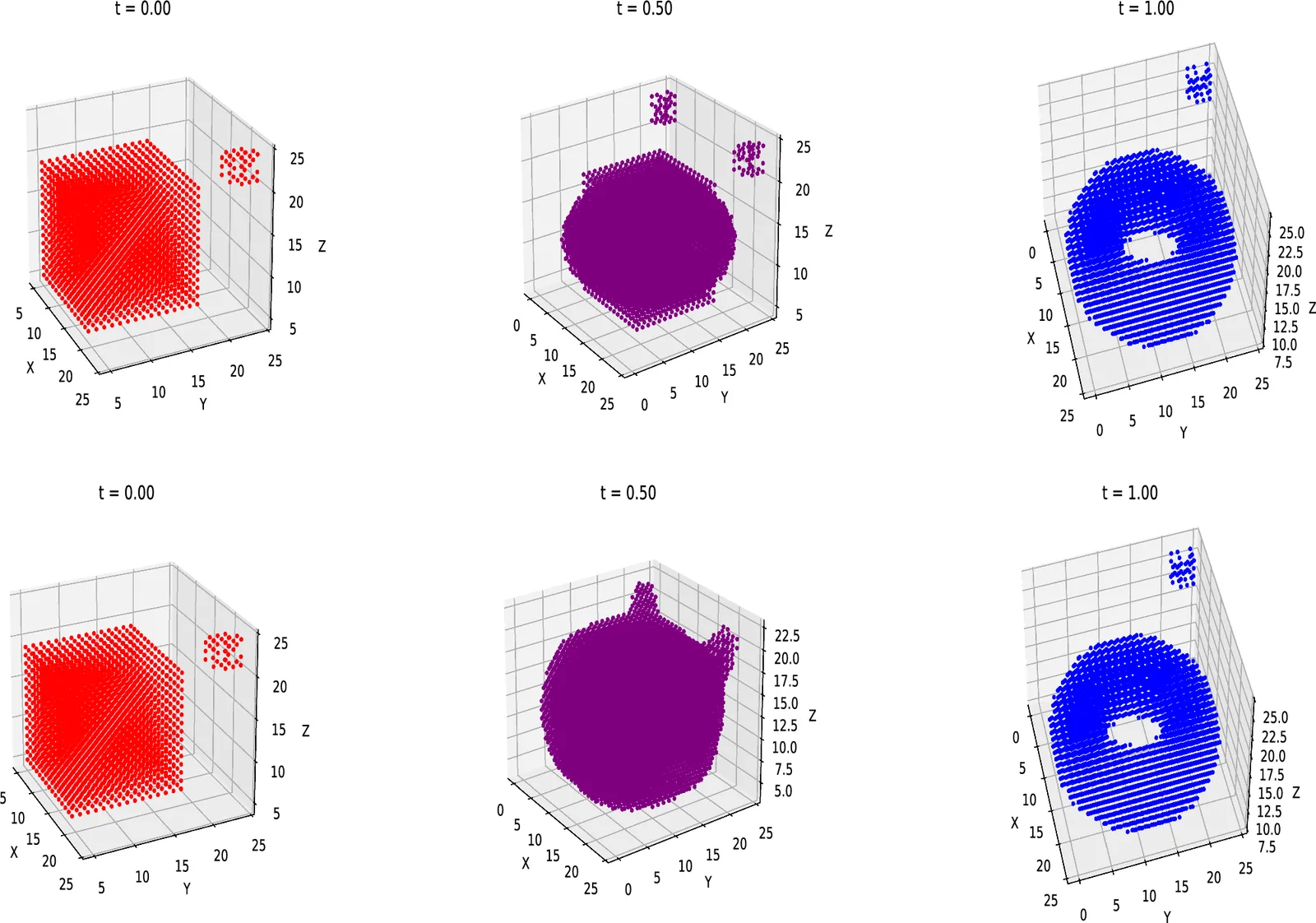
E-ROBOT barycenters via IBP for 3D shapes, for weights t=0,0.5,1: top 3 panels are for λ=0.1 and bottom 3 panels are λ=10). Shapes are on a $22 \times 22 \times 22$ grid. The entropic regularization parameter is ε=0.05

Similar considerations hold for the case of 3D shapes, where we compute the barycenter for values of t=0,0.5,1, between a cube and torus. The original shapes are contaminated by outliers, mimicking the logic as in the 2D case. In Figure 6, we display the results for the E-ROBOT with small λ (three top plots) and the E-ROBOT with large λ (three bottom panels). Also in the 3D case, the key point is that in the E-ROBOT with large λ, the IBP algorithm considers the transport of both the shapes and the outliers, treating them as part of a single, coherent distribution. In contrast, the E-ROBOT with small λ limits the maximum cost of transporting mass, particularly between the far-apart main shapes and the outliers. This is evident comparing the top and bottom middle plots.

### Gradient flows for corrupted 2D shapes

Let $\mu \in \mathcal {P}(X)$ be a probability measure on a compact set $X \subset \mathbb {R}^d$. Gradient flows are the continuous-time analogue of gradient descent: they describe how a probability distribution $\mu _t$ evolves with time *t*. In optimal transport, this evolution takes place in the Wasserstein space of probability measures. Such flows appear naturally in machine learning, for example in generative modeling (evolving a model distribution toward data), domain adaptation (aligning source and target), and clustering/density estimation (regularizing or smoothing empirical measures).

To compare the de-biased robust Sinkhorn divergence $\overline{W}_{\varepsilon ,\lambda }$ with the standard Sinkhorn-type loss for $W_1$, a natural experiment is to let a model distribution $\mu _t$ evolve along the E-ROBOT gradient flow of a data-fit loss that drives it toward a target distribution ( Santambrogio (2015)). This non-parametric fitting setup makes explicit the role of the robustness parameter λ in the presence of outliers. Classical E-OT flows suffer from well-documented artefacts: an entropic shrinkage bias (working on the entropic regularized OT alone pulls μ toward an over-concentrated measure), and – on the other end of the spectrum – kernel MMDs can show vanishing gradients near the support’s extremes; see Feydy et al. (2019). These issues motivate the use of a robust, regularized: $\overline{W}_{\varepsilon ,\lambda }$.

More in detail, recall the E-ROBOT negentropy functional $F_{\varepsilon , \lambda }(\mu )$ in (23). This functional is strictly convex and differentiable; when $\mu =\nu$, the Schrödinger potentials coincide, $\phi ^* = \psi ^*$. Consequently, the (first-variation) gradient of the E-ROBOT negentropy reads $\nabla F_{\varepsilon , \lambda }(\mu ) = -\phi ^*/2,$ and the corresponding Wasserstein gradient flow is ${d\mu _t}/{dt} = -\,\nabla F_{\varepsilon , \lambda }(\mu _t).$ To simulate this continuous-time PDE, one may resort to the explicit (forward) Euler scheme with step size τ>0, where the current distribution is pushed forward by a small displacement along the negative gradient field. If $\mu _k = n^{-1}\sum _{i=1}^n \delta _{x_i^k}$ is an empirical measure at the *k*-th step, this reduces to particle updates of the familiar gradient descent form $x_i^{k+1} = x_i^{k} - \tau \,\nabla _{x_i} F_{\varepsilon , \lambda }(\mu _k),$ for i=1,⋯,n. In Python code (as in the GeomLoss package), one evaluates the loss with parameters $(\varepsilon ,\lambda )$, back-propagates to obtain $\nabla _{x_i}F_{\lambda ,\varepsilon }$, and applies the Euler step above to all particles. The step size τ controls stability and small values of τ imply slower but more faithful to the continuous flow. This explicit discretization exactly mirrors the procedure used in Section 4 of Feydy et al. (2019), which contrasts kernel MMD, biased entropic OT, and the de-biased Sinkhorn divergence on toy registration and gradient flow tasks.

**Fig. 7:**
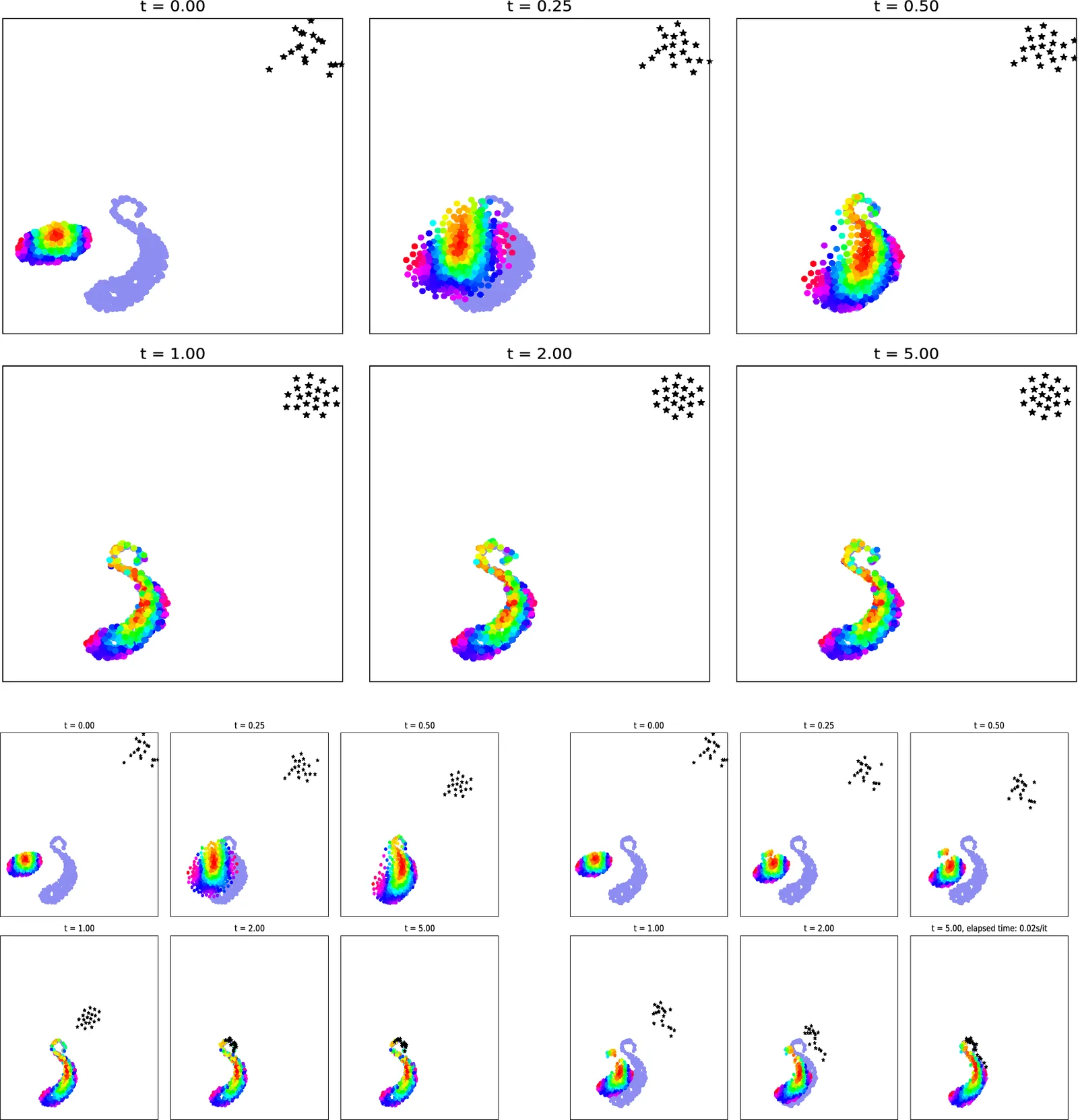
Gradient flows for 2D shapes via entropic regularized OT. Top panels: E-ROBOT with λ=0.6, bottom left 6 panels E-ROBOT with λ=6, and bottom right 6 panels E-OT with $W_2$. The entropic regularization parameter is ε=0.05. The learning rate, i.e. time step τ, is set equal to 0.05, similarly to the default value in GeomLoss

For the sake of visualisation and following the existing examples available in the GeomLoss, we focus on 2D shapes. In Figure 7 we display the gradient flows for the E-ROBOT with a small value of λ (top 6 panels) and a with large λ (bottom panels). The plots showcase how the cost truncation parameter λ controls the E-ROBOT method’s robustness to outliers. For a large value of λ (bottom panels), the E-ROBOT approach behaves similarly to standard E-OT because the cost truncation is effectively removed. In this case, the gradient flow illustrates that the outliers (black stars) are transported and embedded into the central blob of mass as the shapes evolve. This is visually evident as the black stars, initially separate, are drawn into the evolving mass for t≥0.5 (they are getting closer and closer to the blobs). This aspect highlights the sensitivity of standard E-OT to anomalous data points. This is evident also in the bottom panels, which are for the E-OT with $W_2$, as obtained using the command gradient_flow in GeomLoss—which remains the faster approach, as indicated by the time for each iteration. In contrast, for a small λ (top panels), the truncated cost function limits the maximum transport cost between far-apart points. The gradient flow for small λ demonstrates the E-ROBOT method’s robustness: the flow of the main blob proceeds without being influenced by the distant outliers. The gradient flow effectively excludes these outliers from the optimal transport plan, allowing the algorithm to focus on the smooth transformation of the core blobs. This behaviour confirms that the E-ROBOT method successfully mitigates the influence of outliers by preventing them from being part of the transport. However, an important remark is in order. A closer inspection of the gradient flow in the top panels of Figure 7 reveals a subtle but important phenomenon: the outliers are not perfectly stationary from the very beginning. For early timesteps (t=0.25 or t=0.5), they exhibit some minor movement before settling into a fixed configuration. This initial movement is a natural consequence of the gradient descent optimization process. The algorithm, starting from the initial configuration at t=0, is in the process of finding the optimal transport plan. At this early stage, there is a weak but non-zero influence from the distant points (the main blob) on the outliers. The gradient flow is essentially taking small, exploratory steps in the direction of steepest descent for the overall system. However, the core mechanism of the E-ROBOT method – the truncated cost function – quickly takes precedence. The algorithm realises that the cost of moving the outliers is prohibitively high due to the small λ value: this leads to outliers motion becoming negligible and the gradient flow for the outliers effectively vanishes as the algorithm converges on the optimal, outlier-excluding solution.

Referring to the plots in Appendix B, the key conclusion from the experiment is that the use of $\overline{W}_{\lambda ,\varepsilon }$ is preferable to the MMDs for gradient flow. Indeed, $\overline{W}_{\lambda ,\varepsilon }$ provides a more reliable, namely a more geometry aware, flow and more robust gradient signal for moving probability distributions toward each other, especially when they are initially far apart and contain outliers.

### Image color transfer

**Fig. 8:**
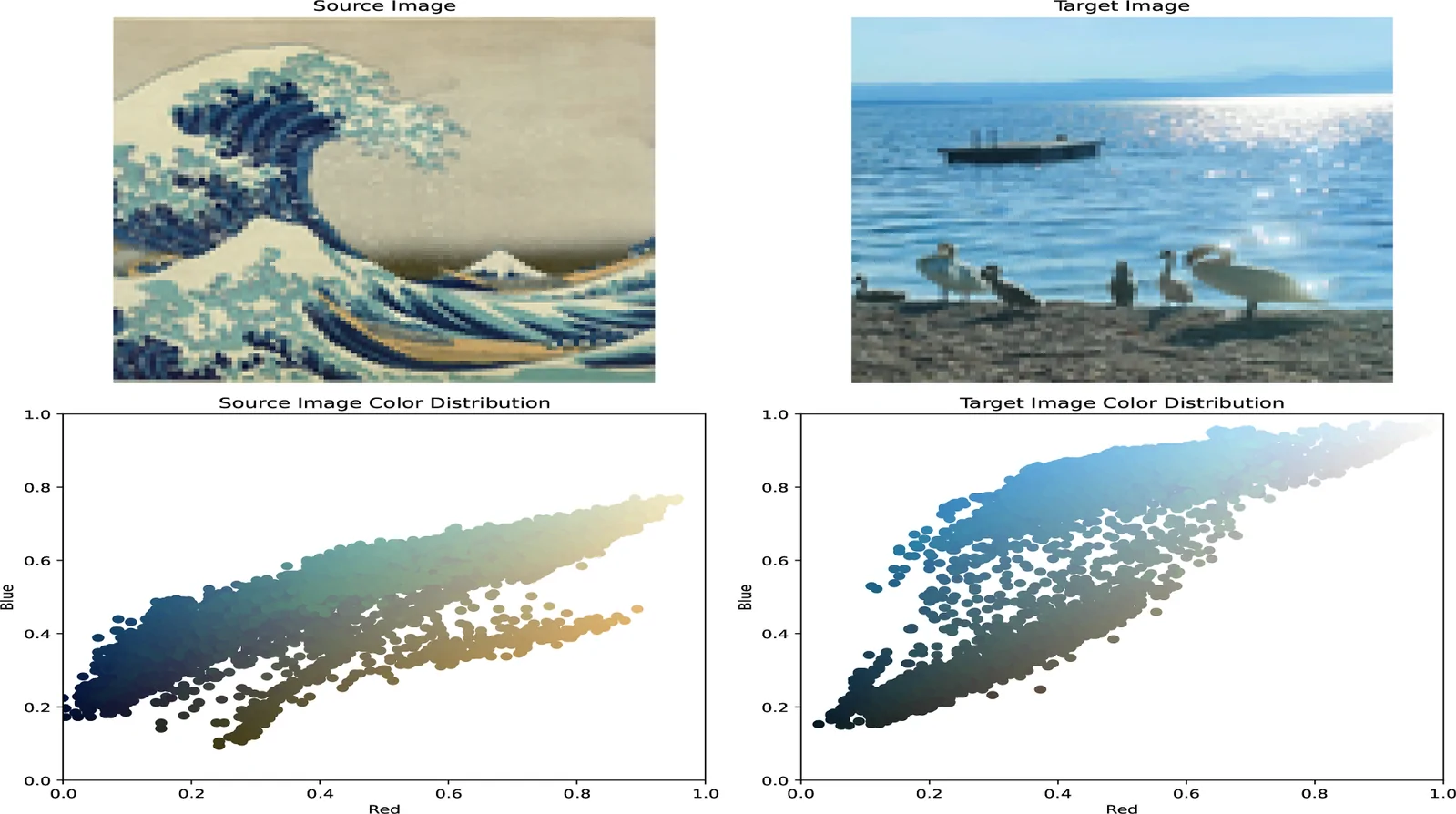
Source (left) and target (right) distributions. Source: *The Great Wave off Kanagawa* by Hokusai, picture credit: Wikipedia. Target: A scene on Lac Léman, Switzerland, picture credit: Nadia La Vecchia

**Fig. 9:**
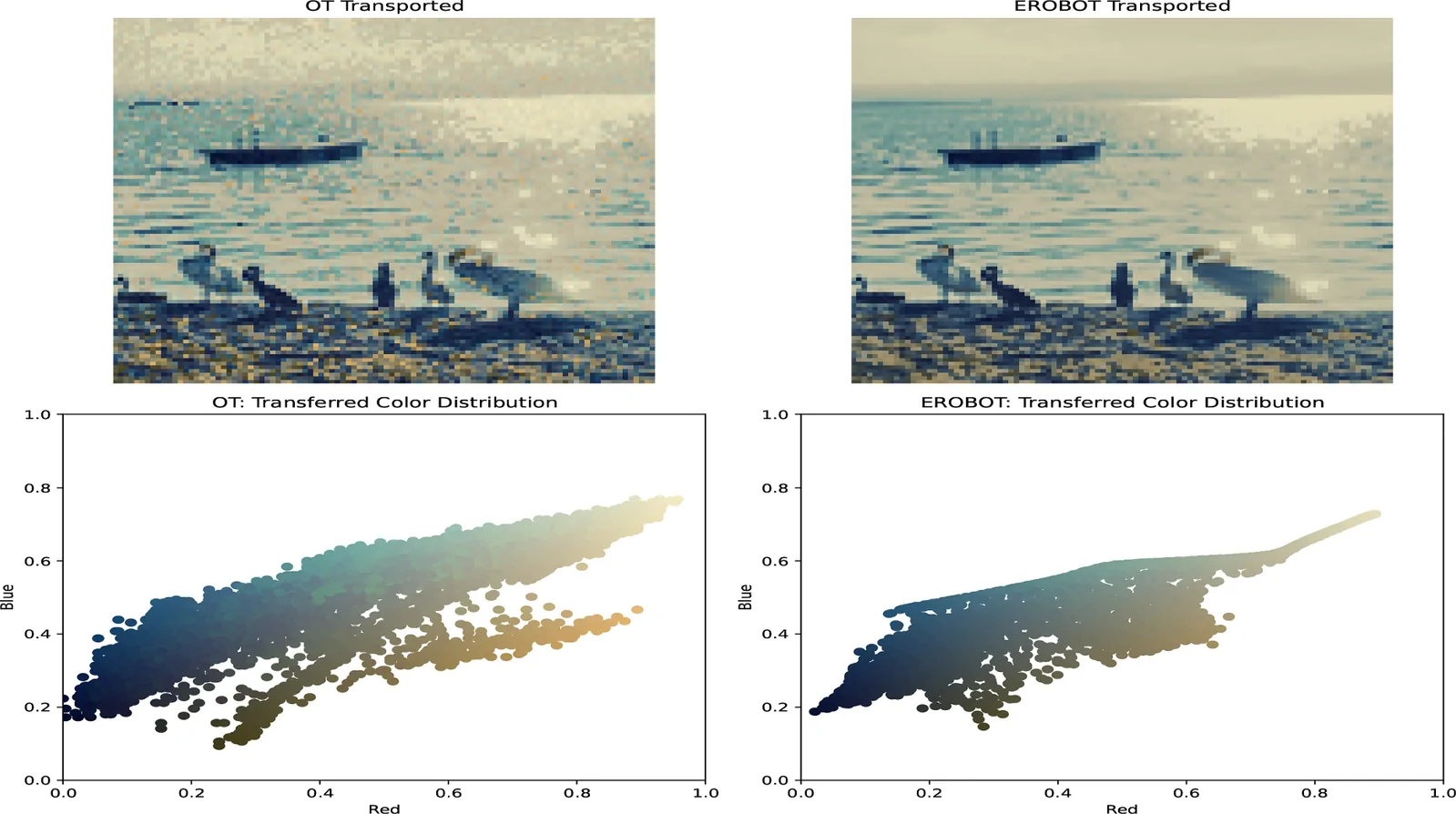
Results of color transfer. Top panel: Output images for OT (left) and E-ROBOT (right), with λ=20 and ε=0.01. Bottom: Their corresponding color distributions

Color transfer is a fundamental and highly applicable task in computer vision and computer graphics. Its importance stems from its role as a core problem in image manipulation and its wide range of practical applications. For instance, it can be used for aesthetic enhancement of photographs and for accessibility purposes, such as modifying color schemes for color-blind viewers or restoring the faded color palette of historical images.

In machine learning, from a computational perspective, color transfer serves as a canonical example of distribution alignment. Indeed, the problem of matching the color distribution of a source image to a target is analogous to aligning the feature distributions of two datasets. Methods that solve color transfer robustly and in large-dimensions, like E-ROBOT, provide valuable insights and tools for this machine learning task. With this regard, it is well-known that the regularization of the transport plan helps to remove colorization artifacts due to noise amplification. More precisely, both OT and ROBOT map between complicated densities can be non smooth. As a result, using directly their transport plans to perform color transfer amplifies the noise in flat areas of the image (namely, it creates colour artifacts). We refer to Ferradans et al. (2014) for a discussion.

We demonstrate the practical efficacy of E-ROBOT on a color transfer task, adapting the palette of Hokusai’s *The Great Wave off Kanagawa* (source image) to that of a scene on Lake Léman (target image), in the Geneva (Switzerland) area; see Figure 8 for the pictures (top panels), with the corresponding color distributions (bottom panels), as obtained using a Red-Blue representation. The ultimate goal is to impose on the target image the colours of Hokusai’s paint, while preserving the smoothness of both images and avoiding the creation of artifacts.

The bottom plots of Figure 8 highlight that the source image contains some colours that pose significant challenges for traditional OT. For instance, there are extreme pigment variations (the vibrant Prussian blue used in the woodblock print creates saturated blue values that fall outside the natural color distribution of the target landscape photograph) or compositional elements (the distinctive white foam splashes and the yellow boat in Hokusai’s work create isolated color clusters that don’t correspond to any elements in the target image).

The results in Figure 9 reveal striking differences in how OT and E-ROBOT handle these challenges. The OT result exhibits notable artifacts, including a speckled noise pattern indicative of unstable pixel assignments, evidenced by a persistent secondary peak in the red region of its distribution. This secondary peak represents OT’s attempt to overfit to red/yellow colours, forcibly matching anomalous source colours to inappropriate target regions. The resulting distribution shows issues in the red channel, where the artificial peak creates unnatural tones and distorted color balances.

In contrast, the E-ROBOT transformation (right panels) demonstrates remarkable robustness. The resulting color distribution illustrates that the robust Sinkhorn divergence effectively downweights the influence of extreme colours. The algorithm recognizes that certain source colours (like the intense Prussian blue or extreme tones of yiellow) entail large transport cost, and thus prevents them from distorting the overall color mapping. This robustness is visually apparent in the transformed image. For instance, the sky tones transition naturally from Hokusai’s distinctive palette to the target’s atmospheric blues without the artificial color casts that plague the OT result via a number of grey untied dots. Moreover, thanks to the regularization, the E-ROBOT features smoothness in the final picture, confirming its statistical superiority for tasks involving real-world images with complex color distributions and inherent outliers.

## Conclusion and possible developments

We introduce the E-ROBOT framework, which combines the outlier robustness of ROBOT with the computational efficiency of entropic regularization via the Schrödinger bridge. The resulting robust Sinkhorn divergence $\overline{W}_{\varepsilon ,\lambda }$ achieves a dimension-free sample complexity of $\mathcal {O}(n^{-1/2})$, overcoming the curse of dimensionality. We demonstrate its applicability in high-dimensional, heavy-tailed settings for tasks like goodness-of-fit testing, barycenter computation, and gradient flows. E-ROBOT can be implemented with simple modifications to existing optimal transport algorithms.

Besides these theoretical and methodological results, other future developments can be envisioned. Hereunder we mention some of them.

(i) *Parametric inference.* The robust Sinkhorn divergence $\overline{W}_{\varepsilon ,\lambda }$ can be applied as loss function to conduct parametric inference in statistics similarly to MKE (see Bassetti et al. (2006)) or in generative modeling (see Genevay et al. (2018)). Specifically, as in Ma et al. (2025), one may define a parametric model as $\{\mu _{\theta }, \ \theta \in \Theta \subset \mathbb {R}^q, \ q\ge 1\}$. Then, the minimum robust Sinkhorn estimator is defined as

$$ \hat{\theta }_n^{\lambda ,\varepsilon } =\underset{\theta \in \Theta }{\operatorname {argmin}} \ \overline{W}_{\varepsilon ,\lambda }\left( \hat{\mu }_n, \mu _\theta \right) . $$

When there is no explicit expression for the probability measure characterizing the parametric model (e.g. in complex generative models), the computation of $\hat{\theta }_n^{\lambda ,\varepsilon }$ can be difficult. To cope with this issue, one may think of using the minimum expected robust Sinkhorn estimator defined as

$$\begin{aligned} \hat{\theta }_{n, m}^{\lambda ,\varepsilon } =\underset{\theta \in \Theta }{\operatorname {argmin}} \ {\mathbb {E}}_m \left[ \overline{W}_{\varepsilon ,\lambda }\left( \hat{\mu }_n, \hat{\mu }_{\theta , m}\right) \right] , \end{aligned}$$

where the expectation $\mathbb {E}_m$ is taken over the distribution $\mu ^{(m)}_{\theta }$. To implement $\hat{\theta }_{n, m}^{\lambda }$ one can rely on Monte Carlo methods and approximate numerically ${\mathbb {E}}_m [\overline{W}_{\varepsilon ,\lambda }\left( \hat{\mu }_n, \hat{\mu }_{\theta , m}\right) ]$. The existence, measurability, and consistency of the resulting estimator should be proved as in Bernton et al. (2019). Moreover, since the sample complexity of $\overline{W}_{\varepsilon ,\lambda }$ scales with $n^{-1/2}$, we conjecture that it is possible to prove root-*n* consistency and asymptotic normality of $\hat{\theta }_{n, m}^{\lambda }$, similarly to the results for $W_2$ in del Barrio and Loubes (2019)—a conjecture that does not make sense for OT- and ROBOT-based estimators in multivariate setting. For the resulting estimators, one may think also of deriving small-sample approximation to their distributions via saddlepoint techniques, exploring connections in the setting of dependent data (as in La Vecchia and Ronchetti (2019); Jiang et al. (2023)) or of independent data (as in La Vecchia et al. (2022)). Moreover, these connections to the saddlepoint techniques can unveil other still unexplored links to econometrics passing through the empirical likelihood method; see Moor et al. (2025).

(ii) *GoF via Bregman-type divergence and MMD.* Following the same logic as the GoF test described in §4.1, one can also define test statistics using either the Hausdorff divergence in (25) or the MMD using $k_{\lambda ,\varepsilon }$. In the case of simple hypothesis, the distribution of these statistics can be obtained via Monte Carlo methods, as in §4.1. Moreover, one may think of considering also composite hypotheses and derive the distribution of the test statistics via bootstrap methods, whose statistical guarantees may be proved building on the results in Theorem 9 and on Klatt et al. (2020). The resulting approach based on $\overline{W}_{\varepsilon ,\lambda }$ may offer an alternative to the recent developments in Hu (2025), where the max-sliced Wasserstein distance is applied.

(iii) *Relaxing the assumptions.* Some of our results of this paper assume compact support: this is common in the OT literature for technical convenience. With this regard, we notice that some of the results in the paper (like Proposition 1, 2, and 3) already hold for noncompact spaces. Thus, one may think of relaxing the compactness assumption—e.g. one may replace it with a tail/tightness assumption. In addition, we highlight that many of our theoretical and methodological results can be extended to the case where we use a generic cost function is $\varepsilon ^{-1} c_{\lambda }$, with $c_{\lambda }=({\tilde{d}}_{\lambda })^p$ and ${\tilde{d}}_{\lambda } = \min (d,2\lambda )$. The use of this cost function could lead to an interesting research topic: extending ${W}_{\varepsilon ,\lambda }$ to ${W}_{\varepsilon ,\lambda , p}$, analogous to the order-*p* Wasserstein distance $W_p$ in classical optimal transport. Both these extensions require modified assumptions and suitable modifications of our proofs available in the Supplementary Material.

(iv) *Selection of* λ *and* ε*.* A key challenge for practitioners is the joint selection of the robustness parameter λ and the entropic regularization parameter ε. We note that this problem is not only open but also fundamentally new, as the literature on OT offers limited guidance even for choosing these parameters individually. The selection of ε in E-OT is often heuristic (see e.g. Goldfeld and Greenewald (2020)), and robust OT methods frequently lack a general data-driven procedure for λ (see e.g. Nietert et al. (2022)). Therefore, the joint calibration required for E-ROBOT operates on new ground and highlights a clear gap in the current literature that we are planning to fill. While beyond the scope of this paper, which introduces and validates the E-ROBOT framework itself, we conjecture that a solution could be based on a concentration inequality derived from the principles in Theorem 9. A closely related open problem concerns rates of convergence. This includes both the behavior of the robust Sinkhorn divergence $\overline{W}_{\varepsilon ,\lambda }$ as $\varepsilon \rightarrow 0$ and the dependence of the $O(n^{-1/2})$ sample-complexity constant on $(\varepsilon ,\lambda )$. Deriving sharp rates is technically challenging under the truncated cost $c_\lambda$, whose linear growth and non-smoothness prevent the use of standard small-noise expansions and detachment arguments developed for smooth costs. Obtaining such rates would require additional analytical tools, such as modified versions of $c_\lambda$ to build on the results in Malamut and Sylvestre (2025), together with a careful analysis of the bias–variance trade-off induced by the parameters. We regard the development of these parameter-selection strategies, as well as the derivation of convergence rates under E-ROBOT’s truncated geometry, as important directions for future work.

(v) *Possible link to differential privacy (DP).* An intriguing research direction arises from Proposition 12, which shows that E-ROBOT *M*-estimators correspond to MLE under a truncated Laplace deconvolution model. Since the Laplace mechanism (see, e.g., Dwork (2014)) is the canonical algorithmic tool for achieving DP, this connection suggests that the E-ROBOT framework may implicitly embed a noise structure closely related to DP perturbations. The bounded robust cost $c_\lambda$ and the Lipschitz-regularized Schrödinger potentials limit the influence of individual datapoints—an essential ingredient for privacy guarantees. This raises the question of whether E- ROBOT–based estimators may enjoy forms of algorithmic stability or approximate DP. Exploring the sensitivity of the E-ROBOT estimator to single-record changes, and studying its relation to DP, could reveal a novel bridge between OT and DP. We believe this can be an exciting direction opened by our method.

## Supplementary Information

Below is the link to the electronic supplementary material.

Supplementary file 1 (pdf 6054 KB)

## Data Availability

No datasets were generated or analysed during the current study.
