# Supplementary material for: E-ROBOT: a dimension-free method for robust statistics and machine learning via Schrödinger bridge
Source: Stat Comput. 2026 Jun 6;36(4):157. doi: 10.1007/s11222-026-10914-9 (PMC13683502; doi:10.1007/s11222-026-10914-9)
Supplement: Supplementary file 1 [file 11222_2026_10914_MOESM1_ESM.pdf]

Supplementary material for: "E-ROBOT: a dimension-free method for robust statistics and machine learning via Schrödinger bridge"

Davide La Vecchia[1*] and Hang Liu[2]

[1*]Geneva School of Economics and Management, University of Geneva, Bld. du Pont d'Arve, Geneva, CH-1211, Switzerland.
[2]Department of Statistics and Finance, School of Management, University of Science and Technology of China, Jinzhai Rd, Hefei, 230026, Anhui Province, China.

*Corresponding author(s). E-mail(s): davide.lavecchia@unige.ch;
Contributing authors: hliu01@ustc.edu.cn;

Appendix A Proofs

A.1 Preliminary lemmas

Let us recall first some basic definitions from Peyré and Cuturi (2019). A symmetric function k (resp., φ) defined on a set $X \times X$ is said to be positive (resp., negative) definite if for any $n \geq 0$, $x_1, \ldots, x_n \in X$, and vector $r \in \mathbb{R}^n$ the following inequality holds:

$$\sum_{i,j=1}^{n} r_i r_j k\left(x_i, x_j\right) \geq 0, \quad \left(\text{resp.} \quad \sum_{i,j=1}^{n} r_i r_j \varphi\left(x_i, x_j\right) \leq 0\right). \tag{A1}$$

The kernel is said to be conditionally positive if positivity only holds in (A1) for zero mean vectors r (i.e. such that $\langle r, 1_n \rangle = 0$), where 1_n is a n-dimensional vector of ones. If k is conditionally positive, one defines the following norm:

$$\|\mu\|_k^2 \stackrel{\text{def.}}{=} \int k(x,y)\mathrm{d}\mu(x)\mathrm{d}\mu(y). \tag{A2}$$

These norms are often referred to as Maximum Mean Discrepancy (MMD). Let us recall that the reproducing kernel Hilbert space (RKHS) $\mathcal{H}_k$ associated with k is

defined as the completion of the linear span of functions $\{k(x,\cdot) : x \in X\}$ under the inner product $\langle f, g\rangle_{\mathcal{H}_k} := \sum_{i,j} \alpha_i \beta_j k(x_i, x_j)$, for $f = \sum_i \alpha_i k(x_i, \cdot)$, $g = \sum_j \beta_j k(x_j, \cdot)$. Moreover, we recall that, according to Sriperumbudur, Fukumizu, and Lanckriet (2011) (Section 3.2, p. 2399), a continuous kernel k on a compact metric space X is *c-universal* if the following hold: (a) $k(x,x) > 0$ for all $x \in X$, (b) there exists an injective feature map $\Phi : X \to \mathcal{H}$ such that $k(x,y) = \langle \Phi(x), \Phi(y)\rangle$. Finally, we recall that a kernel $k : X \times X \to \mathbb{R}$ is said to be *characteristic* if the associated RKHS embedding of probability measures is injective. That is, for any two Borel probability measures μ and ν on X,

$$\mu \neq \nu \quad \Longleftrightarrow \quad \int k(x,\cdot)\, d\mu(x) \neq \int k(x,\cdot)\, d\nu(x).$$

Characteristicness ensures that the RKHS embedding of probability measures is injective, i.e., the kernel can distinguish between different probability distributions.

Lemma 1 *Let c_λ be the ROBOT cost function defined on $X \times X$, with X being a compact subset of $\mathbb{R}^d$. Then for $\epsilon > 0$, the kernel $k_{\epsilon,\lambda}(x,y) = \exp(-c_\lambda(x,y)/\epsilon)$ is positive c-universal and characteristic.*

Proof Let X be compact and assume that $c_\lambda : X \times X \to [0,\infty)$ is continuous, symmetric, and satisfies $c_\lambda(x,y) = 0$ if and only if $x = y$. It is trivial to show that the function induces a kernel c_λ which is conditionally positive definite—indeed, this is obtained by a symmetric truncation of the energy distance kernel, see e.g. Feydy et al. (2019), §1.1. Moreover, the kernel $k_{\epsilon,\lambda}(x,y) = \exp(-c_\lambda(x,y)/\epsilon)$ is continuous and strictly positive on $X \times Y$, since $c_\lambda(x,y) \geq 0$ and $c_\lambda(x,y) = 0$ if and only if $x = y$ implies $k_{\epsilon,\lambda}(x,y) = 1$ if and only if $x = y$, and $k_{\epsilon,\lambda}(x,y) < 1$ otherwise.. So, we define the canonical feature map $\Phi : X \to \mathcal{H}_{k_\epsilon}$ by $\Phi(x) := k_\epsilon(x,\cdot)$. To show that Φ is injective, suppose $x \neq y$. Then $c_\lambda(x,y) > 0$ implies $k_\epsilon(x,y) < 1$, while $k_\epsilon(x,x) = k_\epsilon(y,y) = 1$. Therefore, $k_{\epsilon,\lambda}(x,\cdot) \neq k_\epsilon(y,\cdot)$ in $\mathcal{H}_{k_\epsilon}$, and hence $\Phi(x) \neq \Phi(y)$. Thus, Φ is injective. Next, we verify that the conditions for c-universality are satisfied by $k_{\epsilon,\lambda}$: since $k_{\epsilon,\lambda}(x,x) = \exp(-c_\lambda(x,x)/\epsilon) = \exp(0) = 1$ for all $x \in X$, we have $k_{\epsilon,\lambda}(x,x) > 0$; as shown above, the canonical feature map $\Phi(x) := k_{\epsilon,\lambda}(x,\cdot)$ is injective. Therefore, $k_{\epsilon,\lambda}$ is c-universal, namely, by definition, this means the RKHS $\mathcal{H}_{k_\epsilon}$ is dense in $C(X)$, the space of continuous real-valued functions on X, equipped with the uniform norm. Moreover, as stated still in section 3.2. of Sriperumbudur et al. (2011), when X is compact, c-universality implies that the kernel is characteristic. Hence, the RKHS induced by $k_{\epsilon,\lambda}$ is characteristic and dense in $C(X)$, thus it is positive c-universal. □

Moreover, we state the following

Lemma 2 *Let $X \subset \mathbb{R}^d$ and $c_\lambda : X \times X \to \mathbb{R}$ be the ROBOT cost function. Let $\mu \in \mathcal{P}(X)$ be a probability measure. Then, for $\varepsilon > 0$, the E-ROBOT negentropy $F_{\varepsilon,\lambda}(\mu)$ admits the representation:*

$$\frac{1}{\varepsilon} F_{\varepsilon,\lambda}(\mu) + \frac{1}{2} = \inf_{\xi \in \mathcal{P}(X)} \left\{ \int \ln\left(\frac{d\mu}{d\xi}\right) d\mu + \frac{1}{2}\|\mu\|^2_{k_{\varepsilon,\lambda}} \right\} \tag{A3}$$

Proof The proof follows along the same lines as in Appendix B.5 of Feydy et al. (2019), to which we refer. Here we sketch its main steps, illustrating the pivotal role of c_λ. By definition, the E-ROBOT negentropy is

$$F_{\varepsilon,\lambda}(\mu) := -\frac{1}{2} \inf_{\pi\in\Pi(\mu,\mu)} C_\varepsilon(\mu,\mu,c_\lambda,\pi),$$

where the entropic cost is given by

$$C_\varepsilon(\mu,\mu,c_\lambda,\pi) = \int c_\lambda(x,y)\, d\pi(x,y) + \varepsilon H(\pi\|\mu\otimes\mu).$$

By standard duality results and symmetry of the potentials, we have that for $\mu \equiv \nu \Rightarrow \varphi^* = \psi^* =: f$, this cost admits the dual formulation:

$$C_\varepsilon(\mu,\mu,c_\lambda,\pi) = \sup_{f\in L^1(\mu)} \left\{ 2\int f(x)\, d\mu(x) - \varepsilon \iint \exp\left(\frac{f(x)+f(y)-c_\lambda(x,y)}{\varepsilon}\right) d\mu(x)d\mu(y) + \varepsilon \right\}. \tag{A4}$$

Hence, the negentropy admits the dual formualtion:

$$F_{\varepsilon,\lambda}(\mu) = -\frac{1}{2} \sup_{f\in L^1(\mu)} \left\{ 2\int f(x)\, d\mu(x) - \varepsilon \iint \exp\left(\frac{f(x)+f(y)-c_\lambda(x,y)}{\varepsilon}\right) d\mu(x)d\mu(y) + \varepsilon \right\}.$$

Now, we consider the kernel $k_{\varepsilon,\lambda}(x,y)$ and perform the change of variable

$$f(x) = \varepsilon \ln\left(\frac{d\xi}{d\mu}(x)\right) \quad \Rightarrow \quad d\mu(x) = e^{-f(x)/\varepsilon} d\xi(x). \tag{A5}$$

Then,

$$\int f(x)\, d\mu(x) = \varepsilon \int \ln\left(\frac{d\xi}{d\mu}(x)\right) d\mu(x) = -\varepsilon \int \ln\left(\frac{d\mu}{d\xi}(x)\right) d\mu(x).$$

Next, we compute the term

$$\begin{aligned}\iint \exp\left(\frac{f(x)+f(y)-c_\lambda(x,y)}{\varepsilon}\right) d\mu(x)d\mu(y) &= \iint \exp\left(\frac{f(x)}{\varepsilon}\right) \exp\left(\frac{f(y)}{\varepsilon}\right) k_{\varepsilon,\lambda}(x,y)\, d\mu(x)d\mu(y)\\ &= \iint k_{\varepsilon,\lambda}(x,y)\, d\xi(x)d\xi(y),\end{aligned}$$

where we use (A5). Therefore the dual expression (A4) becomes:

$$F_{\varepsilon,\lambda}(\mu) = -\frac{1}{2} \sup_{\xi\in\mathcal{P}(X)} \left\{ -2\varepsilon \int \ln\left(\frac{d\mu}{d\xi}(x)\right) d\mu(x) - \varepsilon \iint k_{\varepsilon,\lambda}(x,y)\, d\xi(x)d\xi(y) + \varepsilon \right\}.$$

Dividing both sides by ε and rearranging the terms yields:

$$\frac{1}{\varepsilon} F_{\varepsilon,\lambda}(\mu) + \frac{1}{2} = \inf_{\xi\in\mathcal{P}(X)} \left\{ \int \ln\left(\frac{d\mu}{d\xi}(x)\right) d\mu(x) + \frac{1}{2}\iint k_{\varepsilon,\lambda}(x,y)\, d\xi(x)d\xi(y) \right\}.$$

This completes the proof. □

A.2 Proof of Proposition 3

Throughout this section (where we prove Proposition 3) and the next one (where we prove Proposition 4), we work with normalized Schrödinger potentials, namely we fix $\int e^{\psi^*/\varepsilon}\, d\nu = 1$ (or equivalently, we set $1 = \int e^{\phi^*/\varepsilon}\, d\mu$). This is standard in the E-OT literature, see e.g. Nutz and Wiesel (2022). We highlight that in the proof of the proposition, we do not need the compactness assumption.

Proof (i) From Proposition 1, the optimal potentials satisfy the fixed-point equations:

$$\varphi^*(x) = -\varepsilon \ln \int e^{\psi^*(y)-c_\lambda(x,y)/\varepsilon}\, d\nu(y),$$

$$\psi^*(y) = -\varepsilon \ln \int e^{\varphi^*(x)-c_\lambda(x,y)/\varepsilon}\, d\mu(x).$$

Fix $x, x' \in X$. Using the expression for φ^*, we write:

$$|\varphi^*(x) - \varphi^*(x')| = \varepsilon \left| \ln \int e^{\psi^*(y)-c_\lambda(x,y)/\varepsilon}\, d\nu(y) - \ln \int e^{\psi^*(y)-c_\lambda(x',y)/\varepsilon}\, d\nu(y) \right|. \tag{A6}$$

By the mean value theorem for the logarithm and the boundedness of ψ^*, we can bound this difference using the Lipschitz continuity of c_λ in its first argument. Clearly, c_λ is uniformly continuous on $X \times Y$ and Lipschitz, with Lipschitz constant L. Then: $|\varphi^*(x) - \varphi^*(x')| \leq L\|x - x'\|$. A symmetric argument applies to ψ^*, using the analogous expression and the Lipschitz continuity of c_λ in the second variable. Therefore, both φ^* and ψ^* are Lipschitz continuous.

(ii) To prove boundedness, note that the integrals inside the logarithms in (A6) are bounded above and below due to the boundedness of c_λ. Since the exponential of a bounded function is bounded, and the logarithm of a bounded positive function is also bounded, it follows that $\varphi^* \in L^\infty(X)$ and $\psi^* \in L^\infty(Y)$. More precisely, write $u = e^{\varphi^*/\varepsilon}$ and $v = e^{\psi^*/\varepsilon}$. The Schrödinger equations (see Proposition 1) yield

$$u(x) = \frac{1}{\int K(x,y)\, v(y)\, d\nu(y)}, \qquad v(y) = \frac{1}{\int K(x,y)\, u(x)\, d\mu(x)}, \quad K(x,y) = e^{-c_\lambda(x,y)/\varepsilon}.$$

Because $0 \leq c_\lambda \leq 2\lambda$, we have $e^{-2\lambda/\varepsilon} \leq K \leq 1$ for all (x, y). Under the assumed normalization, we have $\int v\, d\nu = 1$, so

$$\int K(x,y)\, v(y)\, d\nu(y) \in \left[e^{-2\lambda/\varepsilon}, 1\right] \quad \Rightarrow \quad u(x) \in \left[1, e^{2\lambda/\varepsilon}\right],$$

namely $0 \leq \varphi^*(x) \leq 2\lambda$ uniformly in x. By symmetry (or by normalizing $\int u\, d\mu = 1$) we also get $0 \leq \psi^*(y) \leq 2\lambda$ uniformly in y. Hence $\varphi^* \in L^\infty(X)$ and $\psi^* \in L^\infty(Y)$. □

A.3 Proof of Proposition 4

Proof From Proposition 1, the Schrödinger potentials satisfy

$$\phi(x) = -\varepsilon \ln \int e^{\psi(y) - \frac{1}{\varepsilon} c_\lambda(x,y)}\, d\nu(y),$$

and

$$\phi_n(x) = -\varepsilon \ln \int e^{\psi_n(y) - \frac{1}{\varepsilon} c_\lambda(x,y)}\, d\nu_n(y).$$

Looking at the integrand, let us define the functions

$$f(x,y) := e^{\psi(y) - \frac{1}{\varepsilon} c_\lambda(x,y)},$$

and

$$f_n(x,y) := e^{\psi_n(y) - \frac{1}{\varepsilon} c_\lambda(x,y)}.$$

We first establish some regularity properties of these functions. Since ψ and ψ_n are uniformly Lipschitz and bounded (by Proposition 3), and c_λ is continuous and bounded, it follows that for each $x \in X$, the maps $y \mapsto f(x, y)$ and $y \mapsto f_n(x, y)$ are uniformly bounded and equicontinuous. The exponential of a bounded Lipschitz function is again bounded and Lipschitz, so both $f(x, \cdot)$ and $f_n(x, \cdot)$ are uniformly Lipschitz in y, uniformly over x. Next, consider the

function class $\mathcal{F} := \{f_x(y) := f(x,y) \mid x \in X\}$. This class is uniformly bounded, uniformly Lipschitz in y, and indexed by the compact set $X \subset \mathbb{R}^d$. As a result, it has finite uniform entropy[1] and is a Glivenko–Cantelli class (see (Van Der Vaart & Wellner, 1996, Section 2.4, Theorem 2.4.1)). Therefore:

$$\sup_{f\in\mathcal{F}} \left| \int f(y)\, d\nu_n(y) - \int f(y)\, d\nu(y) \right| \to 0.$$

This supremum over $\mathcal{F}$ can be rewritten as a supremum over $x \in X$. Indeed, each $f \in \mathcal{F}$ is of the form $f_x(y) = f(x,y)$ for some $x \in X$, and the map $x \mapsto f_x$ is continuous in the uniform topology on $\mathcal{C}(X)$, due to the regularity of ψ and c_λ. Moreover, the class $\mathcal{F}$ is uniformly bounded and equicontinuous, and X is compact. Hence, we can equivalently write:

$$\sup_{f\in\mathcal{F}} \left| \int f(y)\, d\nu_n(y) - \int f(y)\, d\nu(y) \right| = \sup_{x\in X} \left| \int f(x,y)\, d\nu_n(y) - \int f(x,y)\, d\nu(y) \right|.$$

To incorporate the approximating functions $f_n(x,y)$, we observe that $f_n(x,y) \to f(x,y)$ pointwise as $n \to \infty$, due to pointwise convergence $\psi_n(y) \to \psi(y)$ and continuity of c_λ. As established earlier, both $f_n(x,\cdot)$ and $f(x,\cdot)$ are uniformly bounded and equicontinuous in y, uniformly in x, so the family $\{f_n(x,\cdot)\}$ is uniformly integrable. An application the triangle inequality yields

$$\begin{aligned} \left| \int f_n(x,y)\, d\nu_n(y) - \int f(x,y)\, d\nu(y) \right| &\le \left| \int f_n(x,y)\, d\nu_n(y) - \int f(x,y)\, d\nu_n(y) \right| \\ &\quad + \left| \int f(x,y)\, d\nu_n(y) - \int f(x,y)\, d\nu(y) \right|. \end{aligned} \tag{A7}$$

The second term vanishes uniformly in x by the Glivenko–Cantelli result. For the first term, we note that for each fixed $x \in X$, the sequence $f_n(x,\cdot) \to f(x,\cdot)$ converges pointwise in y, and the functions are uniformly bounded and equicontinuous in y, uniformly over x. Therefore, by the dominated convergence theorem, we obtain:

$$\sup_{x\in X} \left| \int f_n(x,y)\, d\nu_n(y) - \int f(x,y)\, d\nu_n(y) \right| \to 0.$$

Since the logarithm is Lipschitz on compact subsets of $(0,\infty)$, and the integrals of $f_n(x,\cdot)$ and $f(x,\cdot)$ are bounded away from zero and infinity, we conclude $\sup_{x\in X} |\phi_n(x) - \phi(x)| \to 0$.

The same argument applies symmetrically to $\psi(y)$ and $\psi_n(y)$ using the fixed-point equations:

$$\psi(y) = -\varepsilon \ln \int e^{\phi(x) - \frac{1}{\varepsilon} c_\lambda(x,y)} d\mu(x), \quad \psi_n(y) = -\varepsilon \ln \int e^{\phi_n(x) - \frac{1}{\varepsilon} c_\lambda(x,y)} d\mu_n(x).$$

Hence, both potentials converge uniformly. □

Remark 1 As noticed by an anonymous referee, an alternative proof of Proposition 4 can be obtained applying the arguments in Nutz and Wiesel (2022). Indeed, under the hypotheses of Proposition 4, one can deduce the uniform boundedness and equicontinuity of the Schrödinger potentials directly from Proposition 3.2 of Nutz and Wiesel (2022), and then proceed by an application of Arzelà–Ascoli theorem. Hence, the proof of Proposition 4 can be streamlined by citing Proposition 3.2 in place of invoking Proposition 3 of our paper at that point.

[1] In this context, finite uniform entropy means that the class $\mathcal{F} = \{f_x(y) := f(x,y) \mid x \in X\}$, where each f_x is Lipschitz and bounded on a compact domain, can be covered by finitely many functions in the $L^2(P)$ norm, uniformly over all probability measures P. Here, the measures P are Borel probability measures on the space X. This ensures that the class supports uniform convergence of empirical integrals. See (Van Der Vaart & Wellner, 1996, Corollary 2.7.10).

Nevertheless, we prefer to retain the current formulation, which makes use of Proposition 3 because this proposition is formulated without compactness assumptions and exploits the specific structure of the truncated ROBOT cost to obtain global Lipschitz and boundedness properties of the potentials on arbitrary $X, Y \subset \mathbb{R}^d$—a result which does not readily follow from Nutz and Wiesel (2022). Hence Proposition 3 provides a conceptual and technical insight about E-ROBOT that would be lost if we only appealed to the assumption of compactness of Proposition 4.

A.4 Proof of Proposition 5

Proof (i) For a fix λ, the associated c_λ is continuous in (x, y) and bounded. Lemma 5.3 in Nutz (2021) implies that, given $\eta > 0$ and $\pi_0 \in \Pi(\mu, \nu)$, there exists $\tilde{\pi} \in \Pi(\mu, \nu)$ such that $\left|\int c_\lambda \, d\tilde{\pi} - \int c_\lambda \, d\pi_0\right| \leq \eta$ and $\frac{d\tilde{\pi}}{d(\mu \otimes \nu)}$ is bounded. So, $\int c_\lambda \, d\tilde{\pi} \leq C_0 + \eta$ and $H(\tilde{\pi} \| \mu \otimes \nu) < \infty$. Thus,

$$C_0 \leq \int c_\lambda \, d\tilde{\pi} + \varepsilon H(\tilde{\pi}|P) \leq C_0 + \eta + \varepsilon H(\tilde{\pi}|P)$$

and (16) follows from the arbitrariness of $\eta > 0$.

(ii) Since, c_λ is lower semicontinuous, the statement follows from Proposition 5.9 in Nutz (2021). □

A.5 Proof of Proposition 6

Proof Since X is compact and c_λ is continuous and bounded on $X \times Y$, the entropic cost $C_\varepsilon(\mu, \nu, c_\lambda, \pi) = \int c_\lambda(x, y) \, d\pi(x, y) + \varepsilon H(\pi \| \mu \otimes \nu)$ is well-defined and finite by assumption. Moreover, it is lower semicontinuous with respect to weak convergence of π, meaning that if $\pi_n \to \pi$ weakly, then

$$\liminf_{n \to \infty} C_\varepsilon(\mu_n, \nu_n, c_\lambda, \pi_n) \geq C_\varepsilon(\mu, \nu, \lambda, \pi).$$

This ensures that the cost of the limiting plan π is not smaller than the limiting cost of the approximating sequence. In particular, it guarantees that any weak limit of a minimizing sequence remains a valid candidate minimizer for the limiting problem. Additionally, the functional is strictly convex in π for fixed $\varepsilon > 0$, due to the strict convexity of the relative entropy term. This guarantees uniqueness of the minimizer π^*, which is crucial for concluding convergence of the entire sequence rather than just a subsequence. Moreover, by Proposition 4, the Schrödinger potentials φ_n^*, ψ_n^* associated with π_n^* converge uniformly to φ^*, ψ^*, the potentials associated with π^*. This implies that the densities

$$\frac{d\pi_n^*}{d(\mu_n \otimes \nu_n)}(x, y) = \exp\left(\varphi_n^*(x) + \psi_n^*(y) - \frac{1}{\varepsilon} c_\lambda(x, y)\right)$$

converge uniformly to the density of π^* with respect to $\mu \otimes \nu$. Since $\pi_n^* \in \mathcal{P}(X \times Y)$ and X, Y are compact, the sequence $\{\pi_n^*\}$ is tight. By Prokhorov's theorem, it admits a weakly convergent subsequence. Let π_∞ be any such limit. Since the marginals $\mu_n \to \mu$ and $\nu_n \to \nu$ weakly, and the potentials converge uniformly, the limit π_∞ must minimize the entropic cost for μ, ν. By uniqueness of the minimizer due to strict convexity, we conclude $\pi_\infty = \pi^*$. Therefore, every subsequence of $\{\pi_n^*\}$ has a further subsequence converging to π^*, which implies that the full sequence converges: $\pi_n^* \to \pi^*$ weakly, as $n \to \infty$. □

A.6 Proof of Proposition 7

Proof Lemma 1 shows that the Lipschitz function c_λ induces, for $\varepsilon > 0$, a positive c-universal kernel $k_{\varepsilon,\lambda}(x, y)$. Moreover, the dual expression in Eq. (14) of the main text as a maximization

of linear forms ensures that $W_{\varepsilon,\lambda}(\mu,\nu)$ is convex with respect to μ and with respect to ν (but not jointly convex if $\varepsilon > 0$). Thus, Proposition 3 and Proposition 4 in Feydy et al. (2019) imply that $\overline{W}_{\varepsilon,\lambda}(\mu,\nu)$ is convex with respect to both inputs. Statements (i), (ii), and (iii) follow from Theorem 1 in Feydy et al. (2019), while (iv) follows from our Proposition 5. □

A.7 Proof of Theorem 9

Proof We aim to bound the quantity: $\mathbb{E}\left[\left|\overline{W}_{\varepsilon,\lambda}(\mu_n,\nu_n) - \overline{W}_{\varepsilon,\lambda}(\mu,\nu)\right|\right]$. To this end, we decompose it as:

$$\left|\overline{W}_{\varepsilon,\lambda}(\mu_n,\nu_n) - \overline{W}_{\varepsilon,\lambda}(\mu,\nu)\right| \le \left|W_{\varepsilon,\lambda}(\mu_n,\nu_n) - W_{\varepsilon,\lambda}(\mu,\nu)\right| + \frac{1}{2}\left|\Delta_n^\mu + \Delta_n^\nu\right| \tag{A8}$$

where $\Delta_n^\mu := W_{\varepsilon,\lambda}(\mu_n,\mu_n) - W_{\varepsilon,\lambda}(\mu,\mu)$, and $\Delta_n^\nu := W_{\varepsilon,\lambda}(\nu_n,\nu_n) - W_{\varepsilon,\lambda}(\nu,\nu)$.

Let φ^*,ψ^* be the optimal Schrödinger potentials for $W_{\varepsilon,\lambda}(\mu,\nu)$. These are bounded, Lipschitz functions due to the regularity of the cost and compactness of the domain—see Proposition 3. Define the suboptimal estimator:

$$\widehat{W}_{\varepsilon,\lambda}(\mu_n,\nu_n) := \int \varphi^*\, d\mu_n + \int \psi^*\, d\nu_n.$$

Then, we consider the identity:

$$\int \varphi_n^*\, d\mu_n + \int \psi_n^*\, d\nu_n =: W_{\varepsilon,\lambda}(\mu_n,\nu_n) = \widehat{W}_{\varepsilon,\lambda}(\mu_n,\nu_n) + \mathrm{bias}_n$$

where

$$\mathrm{bias}_n := W_{\varepsilon,\lambda}(\mu_n,\nu_n) - \widehat{W}_{\varepsilon,\lambda}(\mu_n,\nu_n) = \int (\varphi_n^* - \varphi^*)\, d\mu_n + \int (\psi_n^* - \psi^*)\, d\nu_n.$$

From uniform convergence of Schrödinger potentials stated in Proposition 4, we have $\sup_{x\in X}|\varphi_n^*(x) - \varphi^*(x)| = \mathcal{O}(n^{-1/2})$ and $\sup_{y\in X}|\psi_n^*(y) - \psi^*(y)| = \mathcal{O}(n^{-1/2})$, and since μ_n,ν_n are probability measures, we have the upper bound:

$$\mathbb{E}|\mathrm{bias}_n| \le \sup_x |\varphi_n^*(x) - \varphi^*(x)| + \sup_y |\psi_n^*(y) - \psi^*(y)| = \mathcal{O}(n^{-1/2}).$$

Therefore:

$$\mathbb{E}\left|\widehat{W}_{\varepsilon,\lambda}(\mu_n,\nu_n) - W_{\varepsilon,\lambda}(\mu,\nu)\right| \le \mathbb{E}\left|\int \varphi^*\, d(\mu_n-\mu)\right| + \mathbb{E}\left|\int \psi^*\, d(\nu_n-\nu)\right| + \mathcal{O}(n^{-1/2})$$

Since φ^*,ψ^* are Lipschitz, we apply bounded-Lipschitz norm duality:

$$\mathbb{E}\left|\int \varphi^*\, d\mu_n - \int \varphi^*\, d\mu\right| \le \|\varphi^*\|_{\mathrm{Lip}}\mathbb{E}\|\mu_n-\mu\|_{\mathrm{BL}}, \quad \left|\int \psi^*\, d\nu_n - \int \psi^*\, d\nu\right| \le \|\psi^*\|_{\mathrm{Lip}}\mathbb{E}\|\nu_n-\nu\|_{\mathrm{BL}}, \tag{A9}$$

where the Lipschitz norm of φ^* is defined as:

$$\|\varphi^*\|_{\mathrm{Lip}} := \sup_{x\neq y}\frac{|\varphi^*(x) - \varphi^*(y)|}{d(x,y)},$$

and similarly for ψ^*. From empirical process theory (see e.g. Section 2.1.4, p. 91; 2.2, pp. 95–104; and Section 2.5.1, p. 127] in Van Der Vaart and Wellner (1996)), we have:

$$\mathbb{E}[\|\mu_n-\mu\|_{\mathrm{BL}}] = \mathcal{O}(n^{-1/2}), \quad \mathbb{E}[\|\nu_n-\nu\|_{\mathrm{BL}}] = \mathcal{O}(n^{-1/2}). \tag{A10}$$

Thus, from (A9) and (A10), we have

$$\mathbb{E}\left[\left|\int \varphi^*\, d(\mu_n-\mu)\right|\right] = \mathcal{O}(n^{-1/2}), \quad \mathbb{E}\left[\left|\int \psi^*\, d(\nu_n-\nu)\right|\right] = \mathcal{O}(n^{-1/2}).$$

Hence:

$$\mathbb{E}\left[\left|W_{\varepsilon,\lambda}(\mu_n,\nu_n) - W_{\varepsilon,\lambda}(\mu,\nu)\right|\right] = \mathcal{O}(n^{-1/2}).$$

Similar arguments allow to state: $\mathbb{E}\left[\left|\Delta_n^\mu\right|\right] = \mathcal{O}(n^{-1/2})$, and $\mathbb{E}\left[\left|\Delta_n^\nu\right|\right] = \mathcal{O}(n^{-1/2})$, so, the triangle inequality implies that $\mathbb{E}\left[\left|\Delta_n^\mu + \Delta_n^\nu\right|\right] \le \mathbb{E}\left[\left|\Delta_n^\mu\right|\right] + \mathbb{E}\left[\left|\Delta_n^\nu\right|\right] = \mathcal{O}(n^{-1/2})$. Combining the results into (A8) yields $\mathbb{E}\left[\left|\overline{W}_{\varepsilon,\lambda}(\mu_n,\nu_n) - \overline{W}_{\varepsilon,\lambda}(\mu,\nu)\right|\right] = \mathcal{O}(n^{-1/2})$, as claimed. □

A.8 Proof of Corollary 10

Proof Let μ_n and ν_n be two independent empirical measures, each based on n i.i.d. samples from μ. We aim to bound $\mathbb{E}\left[\overline{W}_{\varepsilon,\lambda}(\mu_n, \mu)\right]$, where the expected value is taken w.r.t. distribution of μ_n. The Sinkhorn loss $\overline{W}_{\varepsilon,\lambda}$ is convex in each argument (Proposition 6). Fixing μ_n, the map $\nu \mapsto \overline{W}_{\varepsilon,\lambda}(\mu_n, \nu)$ is convex. Since $\mathbb{E}[\nu_n] = \mu$, so $\overline{W}_{\varepsilon,\lambda}(\mu_n, \mathbb{E}[\nu_n]) = \overline{W}_{\varepsilon,\lambda}(\mu_n, \mu)$. Then, Jensen's inequality implies $\overline{W}_{\varepsilon,\lambda}(\mu_n, \mu) \leq \mathbb{E}\left[\overline{W}_{\varepsilon,\lambda}(\mu_n, \nu_n)\right]$. Taking expectation over μ_n yields

$$\begin{aligned}\mathbb{E}_{\mu_n}\left[\overline{W}_{\varepsilon,\lambda}(\mu_n, \mu)\right] \leq \mathbb{E}\left[\overline{W}_{\varepsilon,\lambda}(\mu_n, \nu_n)\right] &\leq \mathbb{E}\left[\overline{W}_{\varepsilon,\lambda}(\mu_n, \nu_n)\right] - \mathbb{E}\left[\overline{W}_{\varepsilon,\lambda}(\mu, \mu)\right] \\ &\leq \mathbb{E}\left[\left|\overline{W}_{\varepsilon,\lambda}(\mu_n, \nu_n) - \overline{W}_{\varepsilon,\lambda}(\mu, \mu)\right|\right]\end{aligned}$$

where we make use of $\overline{W}_{\varepsilon,\lambda}(\mu, \mu) = 0$. By Theorem 10, we have

$$\mathbb{E}\left[\left|\overline{W}_{\varepsilon,\lambda}(\mu_n, \nu_n) - \overline{W}_{\varepsilon,\lambda}(\mu, \mu)\right|\right] = \mathcal{O}\left(n^{-1/2}\right),$$

so,

$$\mathbb{E}_{\mu_n}\left[\overline{W}_{\varepsilon,\lambda}(\mu_n, \mu)\right] = \mathcal{O}\left(n^{-1/2}\right).$$

□

A.9 Proof of Proposition 11

Proof The proof is an immediate application of Proposition 5.2 from Rigollet and Weed (2018). We simply need to verify that the E-ROBOT setup fits their general framework. The generative model is $Y = X + Z$, where the noise Z has a known density f with respect to the Lebesgue measure. In the E-ROBOT case, this density is defined by the truncated cost function:

$$f(z) = \frac{1}{\beta} \exp\left(-\frac{1}{\varepsilon} c_\lambda(0, z)\right),$$

where $\beta = \int \exp\left(-\frac{1}{\varepsilon} c_\lambda(0, z)\right) dz$ is the normalization constant. This is a truncated Laplace distribution, a well-defined probability density function because c_λ is bounded and continuous. From Rigollet and Weed (2018), it follows for this noise model, we have:

$$\begin{aligned}W_f(\mu, \nu) &:= \min_{\gamma \in \Pi(\mu,\nu)} \left\{-\int \ln f(x - y) d\gamma(x, y) + H(\gamma \| \mu \otimes \nu)\right\} \\ &= \min_{\gamma \in \Pi(\mu,\nu)} \left\{\int \left(\frac{1}{\varepsilon} c_\lambda(x, y) + \ln \beta\right) d\gamma(x, y) + H(\gamma \| \mu \otimes \nu)\right\} \\ &= \frac{\ln \beta}{\varepsilon} + \frac{1}{\varepsilon} \min_{\gamma \in \Pi(\mu,\nu)} \left\{\int c_\lambda(x, y) d\gamma(x, y) + \varepsilon H(\gamma \| \mu \otimes \nu)\right\} \\ &= C + \frac{1}{\varepsilon} W_{\varepsilon,\lambda}(\mu, \nu),\end{aligned}$$

where $C = \ln \beta$ is a constant independent of μ and ν. Since C and the factor $1/\varepsilon$ are constants with respect to the minimization over $\mu \in \mathcal{P}$, we have:

$$\arg\min_{\mu \in \mathcal{P}} W_f(\mu, \nu_n) = \arg\min_{\mu \in \mathcal{P}} W_{\varepsilon,\lambda}(\mu, \nu_n).$$

From Proposition 5.2 in Rigollet and Weed (2018), under the specified generative model, the maximum-likelihood estimator is such that:

$$\hat{\mu}_n = \arg\min_{\mu \in \mathcal{P}} W_f(\mu, \nu_n). = \arg\min_{\mu \in \mathcal{P}} W_{\varepsilon,\lambda}(\mu, \nu_n).$$

This completes the proof. □

Appendix B Additional numerical exercises

B.1 GoF in the heavy-tailed setting

To complement the analysis presented in Section 1.2 of the main text, we further investigate the goodness-of-fit (GoF) testing problem of Section 4.1 under a pronounced heavy-tailed regime. Specifically, we consider the multivariate t-distribution with one degree of freedom, which represents an extremely heavy-tailed and challenging scenario from a statistical perspective. This setting is well known to strain classical distance-based GoF tests, especially as the ambient dimension increases, since empirical measures become increasingly unstable in the presence of rare but extreme observations.

Our goal is to examine how the proposed statistic $\overline{T}_n$, based on the entropically regularized Sinkhorn divergence $W_{\varepsilon,\lambda}$, performs relative to the W_1-based test when both are subjected to increasing dimensionality and small location shifts. Figure B1 reports the corresponding power curves for dimensions $d = 2$, $d = 15$, and $d = 25$.

The results highlight several important phenomena. First, the test based on W_1 operates close to its nominal level across all dimensions, but its ability to detect the location shift quickly deteriorates as d grows. This is consistent with the well-documented curse of dimensionality affecting Wasserstein distances, where, under heavy tails, the sample complexity required to recover the geometry of the distribution becomes prohibitively large. As a result, even moderate perturbations remain difficult to detect.

In contrast, the statistic $\overline{T}_n$ exhibits markedly stronger and more stable power curves. Across all considered dimensions, $\overline{T}_n$ retains sensitivity to small shifts and adapts more effectively to the presence of extreme values. This robustness can be attributed to the interplay between the truncated cost c_λ and entropic smoothing: the former controls the influence of heavy-tailed observations, while the latter provides additional regularization that stabilizes transport plans and reduces variance. Consequently, $\overline{T}_n$ is able to exploit global geometric deviations even when the sample clouds are strongly dispersed by the heavy tails and the large dimension.

Taken together, these experiments confirm that the E-ROBOT divergence $W_{\varepsilon,\lambda}$ provides a substantial advantage in GoF testing for heavy-tailed distributions. Its performance remains reliable and dimensionally stable, illustrating the benefits of combining entropic regularization with a robust cost function.

B.2 Gradient flow

Arbel, Korba, Salim, and Gretton (2019) propose the use of MMD for gradient flow. Therefore, we repeat the above exercise using three different MMDs: one is obtained using the kernel $k_{\lambda,\varepsilon}$ and two are obtained using a Gaussian kernel, with small and large variance ($\sigma = 0.05$ and $\sigma = 0.65$, respectively). We display the results in Figure B2. Comparing the 6 top plots in Figure 6 of the paper to those obtained via MMDs, we notice that the methods slow down the movement of some points far from the target distribution. This is due to the fact that at these points the kernel values become negligible and this, in turn, implies that the gradients vanish. As a consequence, there

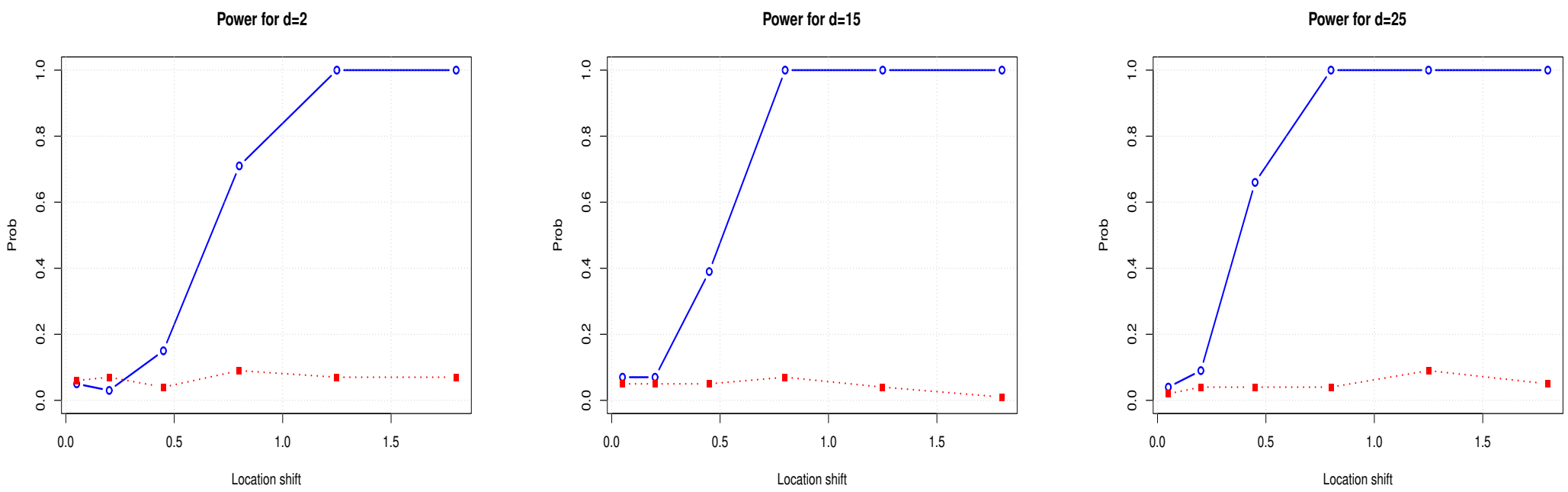

Fig. B1 GoF power curves for testing the null of a d-variate t-distribution with one degree of freedom, location zero, and identity scale matrix. The continuous (blue) curve with circles corresponds to the $\overline{T}_n$ statistic using the entropic regularized Sinkhorn distance with $\varepsilon = 0.05$ and $\lambda = 3$; the dotted (red) curve with squares corresponds to the test based on W_1.

is a region in space where the gradient signals are strong enough to move particles, whereas in other regions there are so weak and they entail no movements. Because of these considerations, we conclude that use of $\overline{W}_{\lambda,\varepsilon}$ is preferable to the MMDs for gradient flow: it provides a more reliable, more geometry aware flow and more robust gradient signal for moving probability distributions toward each other, especially when they are initially far apart and contain outliers.

Different numerical experiments made us understand that the performance of MMDs depends on many aspects of the numerical design, like the type of shapes, their overlapping, and the position of outliers. To elaborate further, we illustrate that MMD-based gradient flow depends on the positions on both the underlying shapes and on the locations of outliers. To this end, we consider the gradient flow between square (source shape) and an oval (target shape), in the presence of outlying values, as depicted in Figure B3.

The numerical experiments in Figure B4 provide a nuanced view of how kernel choice and parameters influence gradient flow performance. The top row demonstrates the effect of the scale parameter ε within the truncated Laplace MMD ($k_{\lambda,\varepsilon}$). A larger ε (e.g., $\varepsilon = 1$, top left) increases the smoothing effect, leading to a more diffuse and stable but potentially less precise flow. Crucially, the flow for the Laplace kernel with a larger scale ($\varepsilon = 1$) demonstrates the most effective overall performance: it successfully merges the central cloud of points into a coherent oval and, despite it transports one outlier star, meaningfully it slowly transports the other outliers towards the target. This effective regularization highlights a potential advantage of this kernel's structure.

The comparison with the Gaussian MMD (bottom row) reveals a more fundamental sensitivity. The Gaussian kernel with a larger bandwidth ($\sigma = 0.55$, bottom left) performs reasonably by preventing vanishing gradients, but it fails to fully resolve the target shape. The flow lacks the necessary precision to fully contract all rainbow square points into a tight oval, and consequently, it also fails to fully integrate the outliers, leaving them stranded. The result is a blurred and incomplete registration.

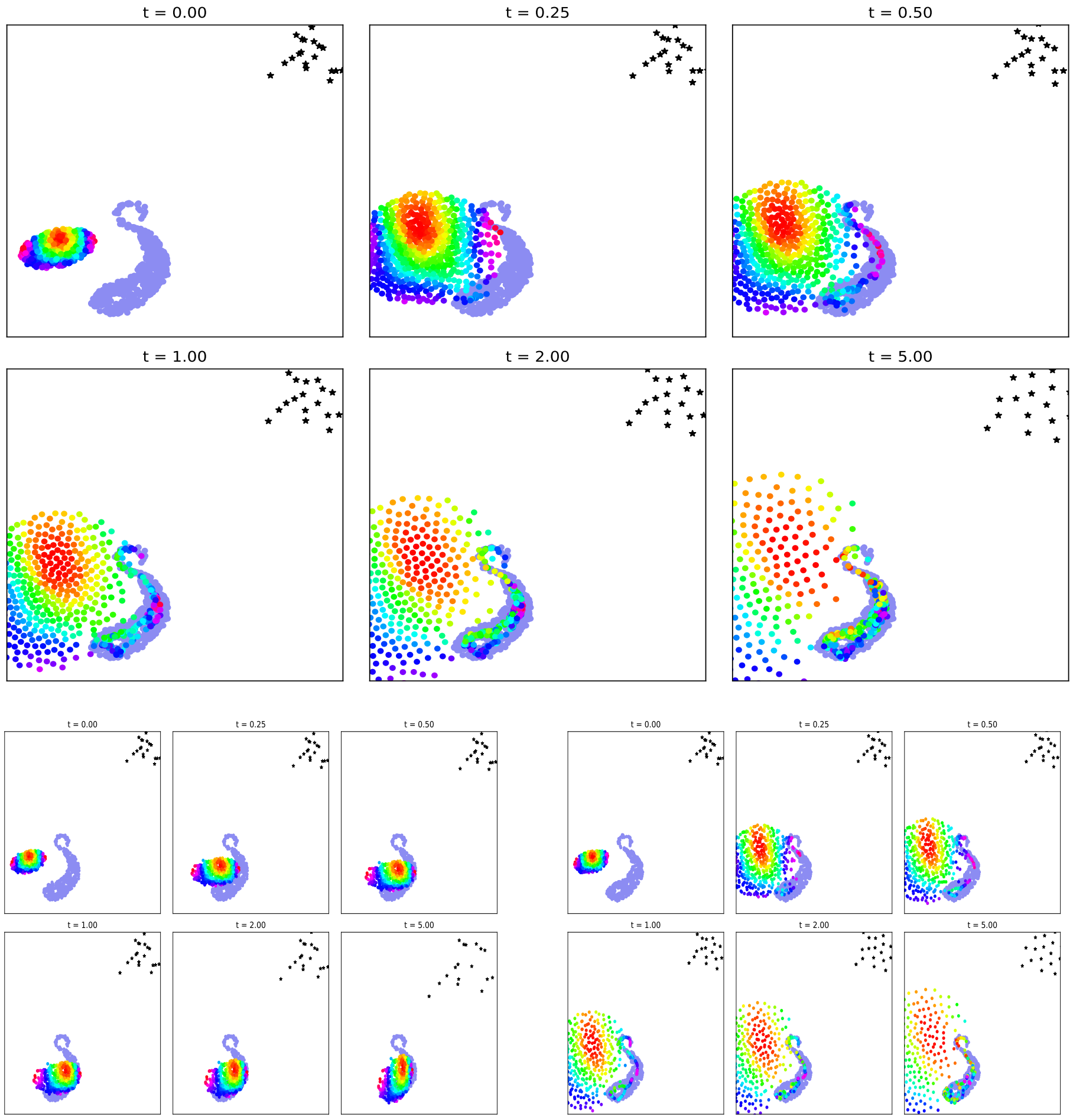

Fig. B2 Gradient flows for 2D shapes via MMDs with different kernels. Top panels: MMD with kernel $k_{\lambda,\varepsilon}$, $\lambda = 0.6$, $\varepsilon = 0.05$ and learning rate, i.e. time step τ, is set equal to 0.05. Bottom left 6 panels: MMD with Gaussian kernel having $\sigma = 0.65$. Bottom right 6 panels: MMD with Gaussian kernel having $\sigma = 0.05$.

The Gaussian with a smaller bandwidth ($\sigma = 0.25$) performs worse, as expected, with severe vanishing gradients stalling the flow for distant points.

These different behaviours underscore a key potential advantage of the truncated Laplace kernel: its parameters λ (robustness) and ε (scale) offer a more interpretable and effective mechanism for balancing smoothness with precision. The Laplace kernel's built-in robustness, which bounds the influence of distant outliers, often makes it a more reliable and easier-to-tune choice than the Gaussian kernel for gradient flow applications, as it provides a more uniform and effective gradient signal across the space.

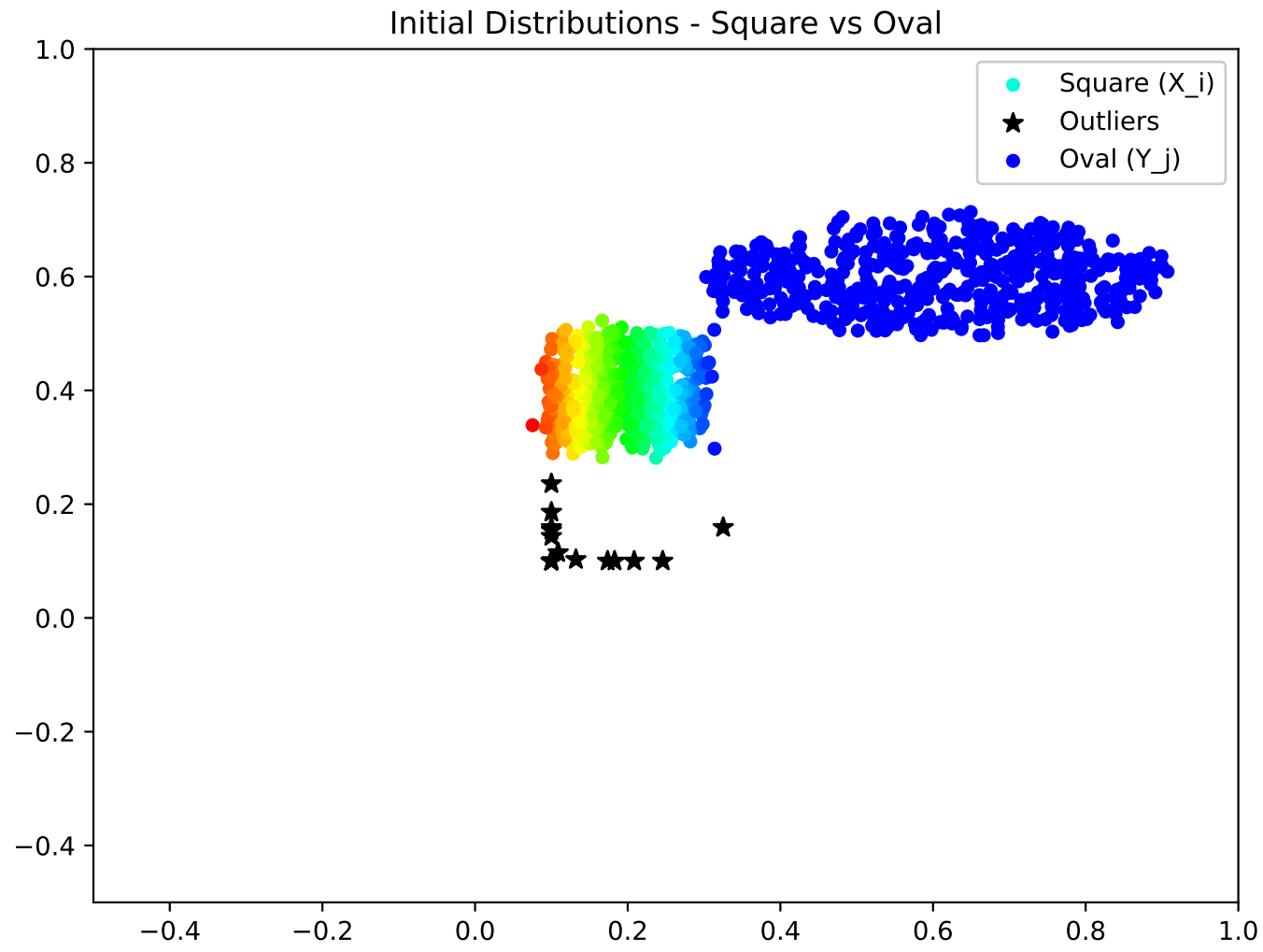

Fig. B3 Square (source shape) and an oval (target shape), in the presence of outlying values.

References

Arbel, M., Korba, A., Salim, A., Gretton, A. (2019). Maximum mean discrepancy gradient flow. *Advances in Neural Information Processing Systems*, *32*, 1-11,

Feydy, J., Séjourné, T., Vialard, F.-X., Amari, S.-i., Trouvé, A., Peyré, G. (2019). Interpolating between optimal transport and MMD using Sinkhorn divergences. *The 22nd international conference on artificial intelligence and statistics* (pp. 2681–2690).

Nutz, M. (2021). *Introduction to entropic optimal transport.* Lecture notes, Columbia University.

Nutz, M., & Wiesel, J. (2022). Entropic optimal transport: Convergence of potentials. *Probability Theory and Related Fields*, *184*(1), 401–424,

Peyré, G., & Cuturi, M. (2019). Computational optimal transport: With applications to data science. *Foundations and Trends® in Machine Learning*, *11*(5-6), 355–607,

Rigollet, P., & Weed, J. (2018). Entropic optimal transport is maximum-likelihood deconvolution. *Comptes Rendus. Mathématique*, *356*(11-12), 1228–1235,

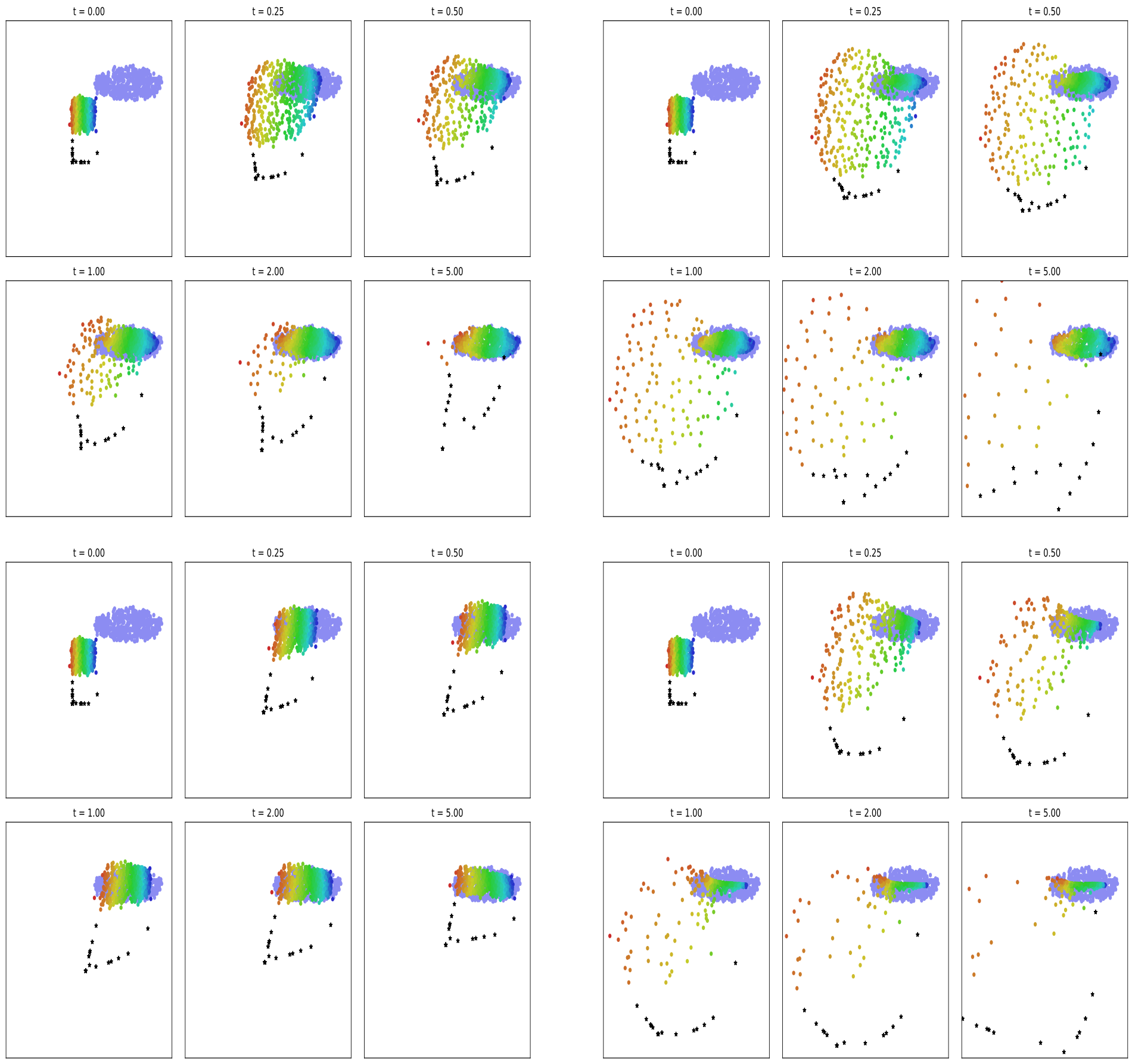

Fig. B4 Gradient flows for 2D shapes via MMDs with different kernels. Top left panels: MMD with kernel $k_{\lambda,\varepsilon}$, $\lambda = 4$, $\varepsilon = 1$. Top right panels: MMD with kernel $k_{\lambda,\varepsilon}$, $\lambda = 4$, $\varepsilon = 0.25$. Bottom left 6 panels: MMD with Gaussian kernel having $\sigma = 0.25$. Bottom left 6 panels: MMD with Gaussian kernel having $\sigma = 0.55$. Bottom left 6 panels: MMD with Gaussian kernel having $\sigma = 0.25$. For all plots the learning rate in the gradient flow is $\tau = 0.05$

Sriperumbudur, B.K., Fukumizu, K., Lanckriet, G.R. (2011). Universality, characteristic kernels and RKHS embedding of measures. *Journal of Machine Learning Research*, *12*(7), ,

Van Der Vaart, A.W., & Wellner, J.A. (1996). *Weak convergence and empirical processes*. Springer.
